# Inferring the Forces Controlling Metaphase Kinetochore Oscillations by Reverse Engineering System Dynamics

**DOI:** 10.1371/journal.pcbi.1004607

**Published:** 2015-11-30

**Authors:** Jonathan W. Armond, Edward F. Harry, Andrew D. McAinsh, Nigel J. Burroughs

**Affiliations:** 1 Warwick Systems Biology Centre and Mathematics Institute, University of Warwick, Coventry, United Kingdom; 2 Molecular Organisation and Assembly in Cells (MOAC) Doctoral Training Centre, University of Warwick, Coventry, United Kingdom; 3 Mechanochemical Cell Biology Building, Division of Biomedical Cell Biology, Warwick Medical School, University of Warwick, Coventry, United Kingdom; Rutgers University, UNITED STATES

## Abstract

Kinetochores are multi-protein complexes that mediate the physical coupling of sister chromatids to spindle microtubule bundles (called kinetochore (K)-fibres) from respective poles. These kinetochore-attached K-fibres generate pushing and pulling forces, which combine with polar ejection forces (PEF) and elastic inter-sister chromatin to govern chromosome movements. Classic experiments in meiotic cells using calibrated micro-needles measured an approximate stall force for a chromosome, but methods that allow the systematic determination of forces acting on a kinetochore in living cells are lacking. Here we report the development of mathematical models that can be fitted (reverse engineered) to high-resolution kinetochore tracking data, thereby estimating the model parameters and allowing us to indirectly compute the (relative) force components (K-fibre, spring force and PEF) acting on individual sister kinetochores in vivo. We applied our methodology to thousands of human kinetochore pair trajectories and report distinct signatures in temporal force profiles during directional switches. We found the K-fibre force to be the dominant force throughout oscillations, and the centromeric spring the smallest although it has the strongest directional switching signature. There is also structure throughout the metaphase plate, with a steeper PEF potential well towards the periphery and a concomitant reduction in plate thickness and oscillation amplitude. This data driven reverse engineering approach is sufficiently flexible to allow fitting of more complex mechanistic models; mathematical models of kinetochore dynamics can therefore be thoroughly tested on experimental data for the first time. Future work will now be able to map out how individual proteins contribute to kinetochore-based force generation and sensing.

## Author Summary

To achieve proper cell division, newly duplicated chromosomes must be segregated into daughter cells with high fidelity. This occurs in mitosis where during the crucial metaphase stage chromosomes are aligned on an imaginary plate, called the metaphase plate. Chromosomes are attached to a structural scaffold—the mitotic spindle, which is composed of dynamic fibres called microtubules—by protein machines called kinetochores. Observation of kinetochores during metaphase reveals they undergo a series of forward and backward movements. The mechanical system generating this oscillatory motion is not well understood. By tracking kinetochores in live cell 3D confocal microscopy and reverse engineering their trajectories we decompose the forces acting on kinetochores into the three main force generating components. Kinetochore dynamics are dominated by K-fibre forces, although changes in the minor spring force over time suggests an important role in controlling directional switching. In addition, we show that the strength of forces can vary both spatially within cells throughout the plate and between cells.

## Introduction

**Fig 1:**
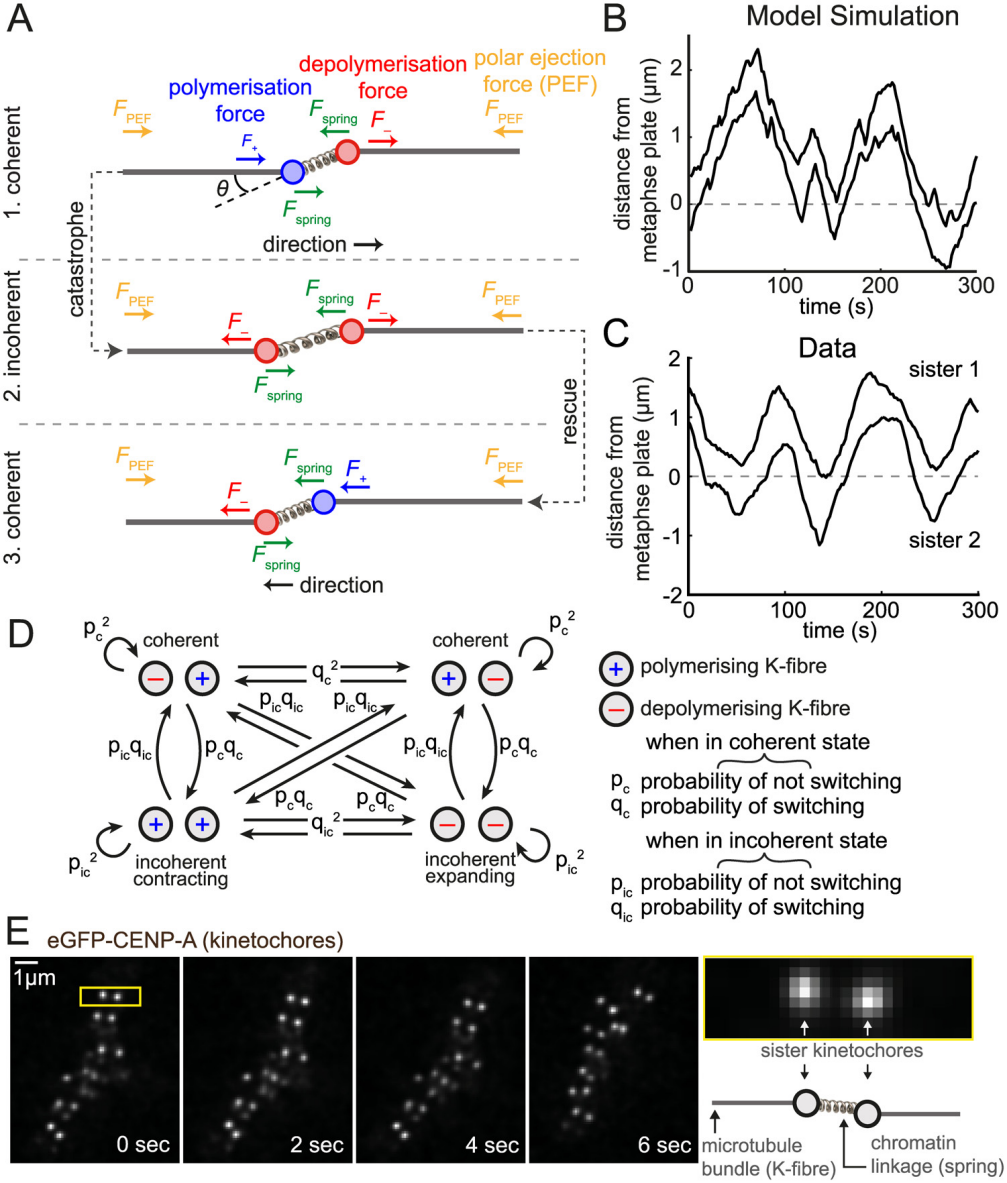
Oscillating stochastic kinetochore model. (A) Model schematic showing the orientation of the two sisters and the respective forces (arrows). Sister kinetochores are connected by a linear spring (producing force *F*_spring_, green) and are attached to a K-fibre in either a polymerising (*F*_+_, blue) or depolymerising (*F*_−_, red) state. The direction of polar ejection forces (PEF, *F*_PEF_) is indicated by orange arrows. Sisters may be off-axis (twist angle *θ*); the spring force (and natural length) are then projected onto the metaphase plate normal. A directional switch is shown with the trailing sister (left) switching first (K-fibre catastrophe), followed by the originally leading sister (K-fibre rescue). See main text for details. (B) Simulated trajectory from model Eq (5) showing the (normal) distance from the metaphase plate of the two sisters. Parameters used were *p*_*c*_ = 0.94, *p*_*ic*_ = 0.61, *v*_+_ = 0.05 μm s^−1^, *v*_−_ = −0.03 μm s^−1^, *L* = 0.8 μm, *κ* = 0.05 s^−1^, *α* = 0.03 s^−1^ and *τ* = 1000 s^2^ μm^−2^, (Δ*t* = 2*s*). See Methods for parameter explanation. (C) Example sister pair trajectory, from kinetochore tracking of live-cell imaging data, showing the (normal) distance from the metaphase plate of the two sisters. (D) The hidden Markov chain transition network for K-fibres switching between polymerising (+) and depolymerising (–) states used in the MCMC algorithm model. *p*_*c*_, *p*_*ic*_ are the probabilities of remaining coherent and incoherent, respectively, and *q*_*c*_ = 1−*p*_*c*_ and *q*_*ic*_ = 1−*p*_*ic*_. (E) Time-lapse sequence of kinetochores. Kinetochores are marked by eGFP-CENP-A. Yellow bordered panel at right shows single kinetochore pair indicated by yellow box in first image.

Chromosomes are attached to, and their movements powered by, kinetochores, multi-protein machines that assemble on each sister chromatid and form dynamic attachments to bundles of kinetochore-microtubules (K-MTs) called K-fibres [1] (see Fig 1A). A long-standing challenge in the mitosis field is to measure the magnitude of forces that kinetochores can generate and identify the molecular components and mechanisms responsible. Nicklas and colleagues addressed this question by using calibrated micro-needles to pull on chromosomes in grasshopper spermatocytes, measuring the force needed to stall chromosome movement [2]. These classic experiments found that > 20 pN was necessary to slow, and 700 pN to stall, chromosome-to-pole movement in anaphase, while there was a much lower stall force (50 pN) for chromosome movement during congression. These measured values are considerably higher than the 0.1 pN that was calculated (based on Stokes law; force = viscosity × chromosome size × velocity) to be required for moving a chromosome under normal conditions [3, 4].

Neither of these approaches, however, are able to separate out the different forces that are acting on a kinetochore: these are known to include (i) K-MT polymerisation and depolymerisation dynamics, (ii) elastic forces from the centromeric chromatin that operates as a compliant linkage between sister kinetochores [5], (iii) polar ejection forces (PEF) mediated by the interactions between non-kinetochore microtubules (MTs) and chromosome arms, (iv) poleward MT flux that involves the continuous displacement of K-fibres towards the pole driven by minus-end depolymerisation and MT sliding [6]. Metaphase provides a unique phase of mitosis for scrutinising these mechanisms since sister kinetochores undergo quasi-periodic oscillatory motion along the spindle axis for several minutes [7, 8]. These movements are possible because each sister kinetochore can adopt either a poleward (P) moving state (the leading sister) by attaching to a depolymerising K-fibre or an away-from-the-pole (AP) state (the trailing sister) by attaching to a polymerising K-fibre. Switching between the AP and P states causes kinetochores to change direction a behaviour termed directional instability [7].

Major advances in understanding these chromosome oscillations have come from tracking fluorescently-labelled kinetochores in living cells [8–13]. In particular, tracking the full complement of kinetochores in three dimensions has generated systematic and comprehensive datasets describing kinetochore motion in human mitotic cells [8, 11] and during mouse meiosis [10]. Such complete kinetochore tracking methodologies have allowed identification of many factors and mechanisms that control different aspects of sister kinetochore dynamics [8, 11, 14–19]. Crucially, it has emerged that in human cells kinetochore oscillations are very stochastic with a wide-range of periods and inter-trajectory variations [8]. Therefore single trajectory analysis is necessary, so that both the unique dynamics inherent to each single trajectory and the inter-kinetochore variation across whole populations can be determined. To achieve this, fitting of kinetochore mathematical models is required, firstly for accurate description of kinetochore dynamics by automated partitioning into P and AP movements, and secondly, in order to unravel the influence of multiple force generators on the dynamics. In particular, through automated analysis large numbers of trajectories can be analysed to determine the trajectory stochasticity and variability; only through model fitting can we obtain quantitative and statistical assessment of the range of dynamics present in trajectories.

Reverse engineering differs from other modelling approaches in cell biology (for a review, see [20]) in that it involves inferring a model (and its parameters) from observed data, a field that is extremely active in many disciplines (e.g., weather prediction, computer vision, epidemiology, phylogenetics, econometrics and financial markets) and has rapidly grown in importance in systems biology over the last decade. It is a leading method for the analysis of microarray data (e.g., BGX software [21]), inference of gene regulatory networks from time series or multiple conditions (e.g., GRENITS software [22]), and biochemical network dynamics [23, 24]. However, there is a dearth of applications to spatial dynamics in cell biology, with only studies of immunological synapse patternation dynamics [25], origin firing in cell division [26], molecular motor force generation in *Drosophila* mitotic spindles [27], and single molecule conformational change [28] employing the methodology to the best of our knowledge. A biophysical model is required for the reverse engineering of stochastic spatial systems, adding to the complexity of an already computationally challenging problem. However, the reverse engineering methodology, particularly in a Bayesian framework, has mature theoretical underpinnings [29]. It is exceptionally powerful and highly flexible with regard to model structure, with an extensive suite of methods for model fitting, including methods for inferring parameters of stochastic differential equations [30]. There are also well-established algorithms for model selection and hypothesis testing [31], which allow biological hypotheses to be addressed in a principled manner, and techniques to incorporate experimental design are also available [32]. These methods are very much under utilised in spatial biological applications.

A key problem however is the choice of the model to fit. There are a number of models of kinetochore oscillations in the literature (see [33, 34] for reviews), with the most biophysically-detailed model [35] demonstrating how oscillations can emerge from dynamic instability of individual K-MTs. However, this model is too complex to fit to individual trajectories since kinetochore positional data is insufficient to identify parameters pertaining to molecular kinetics or energetics. Moreover, the model does not account for additional factors which are very likely crucial *in vivo*, e.g., the interactions between K-MTs in K-fibre bundles, the response of bundles to forces, and the energetic/dynamic effects of K-MT-associated proteins. Therefore, in this paper, we propose a new model that captures the high-level dynamics of the system whilst making minimal assumptions, yet can be directly reverse engineered to individual trajectories. The model is sufficiently simple to allow the determination of all parameters on kinetochore trajectory data alone.

This paper is organised as follows: In section **‘A principle force model of kinetochore oscillations’** we present our new model for kinetochore oscillations and develop the reverse engineering methodology, specifically a Markov chain Monte Carlo (MCMC) algorithm that infers the model parameters (and hidden states) from a trajectory time-series. Model derivation and the model likelihood underpinning the MCMC algorithm are described in the Materials and Methods. In section **‘Reverse engineering individual trajectories’** we demonstrate on a single trajectory how the reverse engineering methodology works, demonstrating high confidence estimation of the model parameters and the switching points of the two sisters. This is extended across 1000s of trajectories in section **‘Identifying oscillatory trajectories using model comparison statistics’**, where we develop techniques that can discriminate the quality of oscillation. In section **‘Trajectory heterogeneity across the metaphase plate’** we examine the variability of trajectories across the population and within cells. In section **‘Relative force components during oscillations’** we show that the K-fibres dominate the mechanics, while there is variation in the PEF across the metaphase plate that explains plate structure and oscillation characteristics throughout the plate. Finally in **‘Discussion’** we discuss the implications of our results to the kinetochore field.

## Materials and Methods

### A stochastic kinetochore model and inference algorithm

In this section we present a new model of kinetochore oscillations, the *coherence-incoherence model*, and an inference algorithm that infers the model parameters from a single paired-sister trajectory time-series.

#### Coherence-incoherence model derivation

To model kinetochore oscillations we consider the action of three forces: the polar ejection force (PEF), the spring constant arising from the centromeric spring connecting the two sisters, and forces due to K-fibres (de)polymerisation. A schematic is shown in Fig 1A, kinetochores moving in 3D. Kinetochore sisters are described by the coordinate pair $\left(X_{t}^{1},X_{t}^{2}\right)$ representing their position normal to the metaphase plate at time *t*, where sister 1 is to the right of sister 2 ($\langle X_{t}^{1}\rangle >\langle X_{t}^{2}\rangle$, 〈⋅〉 indicating time average), and the plate is at *x* = 0. The (projected) inter-sister distance is $X_{t}^{1}-X_{t}^{2}$, and is typically positive but could become negative under an extreme twist into the other two dimensions. This twist will be accounted for through projection. The system is in a high viscous limit [36] so we ignore inertial forces, resulting in the following force balance for kinetochores *k* = 1, 2,

$$F_{\text{drag}}^{k}+F_{\text{K-fibre}}^{k}+F_{\text{spring}}^{k}+F_{\text{PEF}}^{k}+F_{\text{noise}}^{k}=0$$ (1)

where the drag force is proportional to the kinetochore velocity

$$F_{\text{drag}}^{k}=-\gamma \frac{dX_{t}^{k}}{dt}$$

with an (effective) drag coefficient *γ*. The force due to the K-fibre $F_{\text{K-fibre}}^{k}$ is either anti-poleward (polymerising *F*_+_) or poleward (depolymerising *F*_−_). Hence, $F_{\text{K-fibre}}^{k}$ depends on the state of the K-fibre $\sigma_{t}^{k}=\left\{+,-\right\}$. The spring force is assumed to be in the linear regime with the natural length *L* projected by the angle *θ*_*t*_ between the metaphase plate and the sister axis such that

$$F_{\text{spring}}^{k}={\left(-1\right)}^{k}\kappa_{0}\left(X^{1}-X^{2}-L\cos\theta \right)$$

The polar ejection force (PEF) is linearised around the metaphase plate moving the kinetochore towards the plate (as kinetochores approach the poles the PEF becomes nonlinear but near the metaphase plate the linear approximation is applicable [37])

$$F_{\text{PEF}}^{k}=-\alpha_{0}X^{k}$$

*κ*_0_ is the spring constant and *α*_0_ parametrises the linear PEF dependence on position. Finally, $F_{\text{noise}}^{k}$ is a random force due to molecular collisions and would result in Brownian motion of the kinetochore if it was unconstrained. However, there is also likely to be an active component to the noise coming from, for instance fluctuations in the mechanical forces from the K-fibre (de)polymerisation, i.e., the noise is not Brownian in the physical sense.

Substituting the forces into Eq (1) gives the following stochastic differential equation

$$\gamma \frac{dX_{t}^{k}}{dt}={\left(-1\right)}^{k}F_{\sigma_{t}^{k}}+{\left(-1\right)}^{k}\kappa_{0}\left(X_{t}^{1}-X_{t}^{2}-L\cos\theta_{t}\right)-\alpha_{0}X_{t}^{k}+\sqrt{2\gamma k_{B}T_{\text{eff}}}\zeta_{t}$$ (2)

constituting forces from the K-fibre, spring, PEF and random noise, respectively. The K-fibre force term has a minus sign for sister 1 because it lies towards positive infinity, the polymerising and depolymerising K-fibre forces then have signs *F*_+_ > 0 or *F*_−_ < 0, respectively. Here $\sigma_{t}^{k}\in \left\{+,-\right\}$ is the state of the K-fibre attached to sister *k* at time *t* (+ or –, for polymerising or depolymerising, respectively), *k*_*B*_ is Boltzmann’s constant, *T*_eff_ the effective temperature (in Kelvin and most likely inflated due to non-thermal noise from mechanical and ATP-driven fluctuations) and *ζ*_*t*_ is white noise (time derivative of Wiener noise, $\zeta_{t}=\frac{dW_{t}}{dt}$) satisfying 〈*ζ*_*t*_〉 = 0 and 〈*ζ*_*t*_ *ζ*_*t*′_〉 = *δ*(*t*−*t*′). The coefficient of *ζ*_*t*_ is determined by the Einstein relation, a special case of the fluctuation-dissipation theorem [38].

The drag coefficient is extremely difficult to measure or estimate; hence we do not attempt to separate it out as to do so would propagate its large variance to all parameter estimates. We absorb it into our parameters giving,

$$\frac{dX_{t}^{k}}{dt}={\left(-1\right)}^{k}\gamma^{-1}F_{\sigma_{t}^{k}}+{\left(-1\right)}^{k}\kappa \left(X_{t}^{1}-X_{t}^{2}-L\cos\theta_{i}\right)-\alpha X_{t}^{k}+\sqrt{\frac{2k_{B}T_{\text{eff}}}{\gamma }}\zeta_{t}$$ (3)

where κ = κ_0_/γ, α = α_0_/γ. We observe that all terms of Eq (3) have dimensions of speed, in particular $\gamma^{-1}F_{\sigma_{t}^{k}}$ is a velocity, and in absence of the other forces the kinetochore would move at this speed. We define *v*_±_ = *γ* ^−1^ *F*_±_, thus parameterising the action of the K-fibre by an effective velocity. The system thus has two speeds *v*_±_, and two ‘force parameters’, *κ* (s^−1^) and *α* (s^−1^).

Finally, since position measurements are made every Δ*t*, we discretise Eq (3) over time steps of Δ*t*. The forces are likely to be slowly varying over the time interval such that $\int_{t}^{t+\Delta t}F\left(X_{t}^{k}\right)\;dt=F\left(X_{t}^{k}\right)\Delta t$, accurate to $O(\Delta t^{2})$. Integrating over the time interval [*t*, *t* + Δ*t*] we obtain the approximate displacement over a time interval Δ*t*,

$$X_{t+\Delta t}^{k}=X_{t}^{k}+{\left(-1\right)}^{k}v_{\sigma_{t}^{k}}\Delta t+{\left(-1\right)}^{k}\kappa \Delta t\left(X_{t}^{1}-X_{t}^{2}-L\cos\theta_{t}\right)-\alpha \Delta tX_{t}^{k}+\Delta tN\left(0,s^{2}\right)$$ (4)

where, since Wiener noise over an interval is Gaussian (*W*_*t*+Δ*t*_−*W*_*t*_ ∼ *N*(0, ∣Δ*t*∣)), we have defined *s* ^2^ = 2*k*_*B*_ *T*_eff_/(*γ*Δ*t*). Again, *s* has dimensions of speed (e.g., Einstein’s relation would give γ = *k*_*B*_ *T*_eff_/*D*_eff_, where *D*_eff_ is the effective diffusion coefficient of the kinetochore). Retaining the parametrisation in terms of speeds is useful since it is physically meaningful, and thus we quote in the following results with dimensions appropriate for speeds.

Finally, our model for frame to frame kinetochore sister displacements is the following velocity balance equations

$$\begin{array}{cc} (X_{t+\Delta t}^{1}-X_{t}^{1})/\Delta t & =-v_{\sigma_{t}^{1}}-\kappa \left(X_{t}^{1}-X_{t}^{2}-L\cos\theta_{t}\right)-\alpha X_{t}^{1}+N\left(0,s^{2}\right)\\ (X_{t+\Delta t}^{2}-X_{t}^{2})/\Delta t & =+v_{\sigma_{t}^{2}}-\kappa \left(X_{t}^{2}-X_{t}^{1}+L\cos\theta_{t}\right)-\alpha X_{t}^{2}+N\left(0,s^{2}\right) \end{array}$$ (5)

We chose a simple model for the switching of sister state $\sigma_{t}^{k}$ that encompasses as special cases many of the switching choreographies we are interested in. Specifically, the sisters switch direction independently with a probability of switching per frame depending on the state of the other sister (see Fig 1D). The sister pair state can either be coherent (both moving the same direction, i.e., σ_*t*_ = (+, −) or (−, +)) or incoherent (moving in opposite directions, either as an expanding, σ_*t*_ = (−, −), or contracting state, σ_*t*_ = (+, +)). For the MCMC it is more natural to define the probability (per frame) of remaining coherent *p*_*c*_ and the probability (per frame) of remaining incoherent *p*_*ic*_, giving the switching model,

$$\pi \left(\sigma_{t+1}^{k}=\sigma_{t}^{k}|\sigma_{t}^{1}\neq \sigma_{t}^{2}\right)=p_{c},\;\pi \left(\sigma_{t+1}^{k}=\sigma_{t}^{k}|\sigma_{t}^{1}\;=\sigma_{t}^{2}\right)=p_{ic}$$ (6)

Thus, $\sigma_{i}^{k}$ are a hidden Markov chain (hMC) where the state switching rate depends on whether the sisters are coherent.

The model was simulated (Fig 1B) by firstly simulating the sister states according to Eq (6), and then simulating displacements as per Eq (5). We simulated in 1D, i.e., *θ*_*i*_ = 0 for all *i*, producing trajectories that are qualitatively similar to those observed experimentally. These kinetochore like oscillations require *p*_*c*_ > *p*_*ic*_ such that coherent periods are longer than incoherent periods.

#### Model likelihood and reverse engineering MCMC algorithm

We use a Bayesian methodology implemented using a Markov chain Monte Carlo (MCMC) algorithm to compute the posterior probability $\pi (v_{\pm },\kappa ,L,\alpha ,\tau ,\sigma_{t}^{k}|x_{t}^{k},\theta_{t})$ of the parameters and the hidden states ($\sigma_{t}^{k}$) given the observed data $\left(x_{t}^{1},x_{t}^{2},\theta_{t}\right)$, where we use the precision *τ* = *s* ^−2^ to parametrise the noise. The data are measured at equally spaced time points (*t*_*i*+1_−*t*_*i*_ = Δ*t*), frames indexed by *i* = 1, 2,…,*n*. From the (posterior) distribution we can compute all required moments, e.g., mean, variance, and locate the switching times. The model is fitted to each individual pair of sister trajectories separately. In the following we define inter-sister distance $d_{i}=x_{i}^{1}-x_{i}^{2}$ and kinetochore frame displacement $dx_{i}^{k}=x_{i+1}^{k}-x_{i}^{k}$.

We assume trajectories contain no missing data (trajectories are truncated to exclude untracked time points). The likelihood, given the data and the hidden states, is

$$\begin{array}{cc} \pi (x_{i}^{k}|v_{\pm },\kappa ,L,\alpha ,\tau ,\sigma_{i}^{k},\theta_{i}) & =\tau^{n-1}\prod_{k=1,2}\exp(-\frac{\tau }{2}\sum_{i=1}^{n-1}{\left(dx_{i}^{k}-\Delta t\;f_{i}^{k}\left(\sigma_{i}^{k}\right)\right)}^{2})\\ f_{i}^{k}\left(\sigma_{i}^{k}\right) & ={\left(-1\right)}^{k}v_{\sigma_{i}^{k}}+{\left(-1\right)}^{k}\kappa \left(d_{i}-L\cos\left(\theta_{i}\right)\right)-\alpha x_{i}^{k}, \end{array}$$ (7)

For simplicity, in the following we absorb Δ*t* into the parameters *v*_±_, *κ*, *α*; time is thus measured in frames. Here $f_{i}^{k}$ is proportional to the force on sister *k* at frame *i* up to a factor of *γ*.

The posterior probability follows from Eq (7) using Bayes theorem, and is given, up to proportionality, by

$$\begin{array}{cc} \pi (v_{\pm },\kappa ,L,\alpha ,\tau ,\sigma_{i}^{k}|x_{i}^{k},\theta_{i})\propto & \pi_{\alpha }\pi_{v_{+}}\pi_{v_{-}}\pi_{\tau }\pi_{\kappa }\pi_{L}\\ & p_{c}^{\alpha_{c}+N_{c}-K_{c}-1}{\left(1-p_{c}\right)}^{\beta_{c}+K_{c}-1}p_{ic}^{\alpha_{ic}+N_{ic}-K_{ic}-1}{\left(1-p_{ic}\right)}^{\beta_{ic}+K_{ic}-1}\\ & \tau^{n-1}\prod_{k=1,2}\exp(-\frac{\tau }{2}\sum_{i=1}^{n-1}{\left(x_{i+1}^{k}-x_{i}^{k}-f_{i}^{k}\left(\sigma_{i}^{k}\right)\right)}^{2}) \end{array}$$ (8)

comprising the priors, the hidden Markov chain (hMC) and the likelihood, respectively by line. Here *N*_*c*_, *K*_*c*_ are the number of coherence time points and the number of switches to incoherence respectively, and similarly *N*_*ic*_ and *K*_*ic*_ for incoherence and switches to coherence. Naturally, *N*_*c*_ + *N*_*ic*_ = *n*−1 where *n*−1 is the number of displacements. We used conjugate priors as follows: $v_{\pm }∼N\left(\pm \mu_{v},s_{v}^{2}\right)$, *τ* ∼ Γ(*c*, *d*), $\kappa ∼N(\mu_{\kappa },s_{\kappa }^{2})$, $L∼N(\mu_{L},s_{L}^{2})$, $\alpha ∼N(\mu_{\alpha },s_{\alpha }^{2})$ and a Beta distribution on the hMC chain transition probabilities, parametrised by the probability of remaining in the same state per frame, *p*_*c*_ ∼ Beta(*α*_c_, *β*_c_) while the sisters are coherent, *p*_*ic*_ ∼ Beta(*α*_ic_, *β*_ic_) while incoherent. We used weak priors (see Table 1 in S1 Text), except for *p*_*c*_, *p*_*ic*_ for which we used a prior that shifts the probability of no change per frame away from zero, and for *L* we used a prior informed from nocodazole-treated cells (wherein K-MTs are completely depolymerised; S1A Fig; see section 3.1 in S1 Text for Methods). This prior on *L* was required since the model has an *a posteriori* identifiability problem (see section 1.3 in S1 Text).

#### Event detection

The samples for the hidden states $\sigma_{i}^{k}$ were processed for runs of coherence which we defined as a sequence of time-points remaining in the same state (+/– or –/+), but allowing transient changes from that state provided the change lasted for no more than 3 consecutive time points. Furthermore, a coherent run had to contain at least 5 consecutive time points in the same coherent state. We call the beginning and end of a coherent run start and end events, respectively. Each sample *r*, comprising a vector of hidden states ${\left(\sigma_{i}^{k}\right)}^{r}$ for sister *k*, frame *i*, from the MCMC chain was analysed for coherent runs. At each frame *i* we thereby determined a probability of start or end events based on the proportion of samples for which events occurred.

#### Force inference

The force components can be separated into those that use the observed kinetochore location (spring force and PEF), and thus are defined at each time point, and the inferred K-fibre state (polymerisation/depolymerisation) which is defined for the following displacement. By using the inferred model parameters, these components can be estimated. To combine these forces, we use the K-fibre state for the following displacement as an estimate of the K-fibre state at that time point.

### Model selection

In order to objectively identify oscillatory trajectories, we used three methods. Firstly we used Bayes factors (ratio of model marginal likelihoods), comparing the coherence-incoherence model *M*_coh_ to a Brownian motion (BM) model *M*_BM_ that considers both kinetochores to move as independent (1D) BMs (similarly discretised to frames, see section 2.1 in S1 Text, and section 2.2 for more complex BM models). We used Chen’s method [39] to estimate the marginal likelihood *π*(**X**∣*M*) from MCMC samples (see section 2.3 in S1 Text). The data supports model *M*_1_ over *M*_2_ when the Bayes factor *B*[*M*_1_/*M*_2_] = π(**X**|*M*_1_)/π(**X**|*M*_2_) is greater than 1. Secondly, we used an intrinsic model statistic, explained variance (EV), which measures how much of the variance in the kinetochore pair trajectory is explained by the model (see section 2.4 in S1 Text). A pure BM (*M*_BM_) would have a low EV, while a coupled kinetochore pair undergoing saw-tooth oscillation would have an EV of 1. These two methods are based on explaining kinetochore displacements between frames. However, displacements can be arbitrarily reordered; hence these statistics do not account for correlations between consecutive frames as is seen in processive movement. Therefore, our third method is a directional switching statistic designed to account for correlations between displacements by testing if the number of times the direction of motion is changed is consistent with a random walk, where the number of directional switches is binomially distributed (see section 2.5 in S1 Text).

### Live cell imaging and kinetochore tracking

Live-cell imaging and tracking of kinetochores using HeLa-Kyoto (K) cells stably expressing eGFP-CENP-A / eGFP-Centrin1 is described in S1 Text and based on previous work [40, 41]. The core of the tracking software (MATLAB kinetochore tracking software (KiT)) is available from http://mechanochemistry.org/mcainsh/software.php and will be described in a forthcoming paper.

## Results

### A principle force model of kinetochore oscillations

To investigate sister kinetochore dynamics during metaphase we developed a dynamic mathematical model which incorporates the three principle forces acting on kinetochores: a constant driving force from either a polymerising or depolymerising K-fibre, the spring tension in the connecting chromatin spring (modelled as a Hookean spring), and the PEF, linearised around the metaphase plate (Fig 1A, see Materials and Methods), with frame to frame displacement dynamics in Eq (5). Simulations of this coherence-incoherence model are qualitatively consistent with the data (see simulation and data in Fig 1B and 1C). We defined sisters as moving coherently if both sisters are moving in the same direction, i.e. one sister is attached to a polymerising K-fibre (+ state) and the other to a depolymerising K-fibre (– state); otherwise the sisters are in an incoherent state that can be either a +/+ (both polymerising hereafter called contracting) or –/– (both depolymerising hereafter called expanding). Note that, by coherence we refer to coherence *between* K-fibres, rather than to (in)coherence observed *within* K-fibres [19, 42]. In this model sisters switch from + to − (and vice versa) independently through a probabilistic waiting time (exponentially distributed, i.e., there is no location or history dependence), with a waiting time that is dependent on the sister coherence state (Fig 1D). This model produces qualitatively realistic oscillations when the coherence state is longer lived than the incoherence state, i.e., when coherence is restored quickly, either as a sustained switching (both sisters switch direction) or as a switching reversal (first sister switching back to its original direction of motion). Crucially, the sister which switches is chosen at random, i.e., there is no intrinsic bias in the model thereby allowing the bias inherent to the kinetochores to be estimated from the data.

We developed a reverse engineering algorithm for this model within a Bayesian framework, i.e., we used a Markov chain Monte Carlo (MCMC) algorithm to infer the posterior probability $\pi (\Theta ,\sigma_{t}^{k}|X_{t}^{k})$ for the parameters **Θ** (spring constant *κ*; spring natural length *L*; polymerisation *v*_+_, and depolymerisation *v*_−_ speeds; PEF strength *α* and noise *σ* ^2^) and the unobserved sister states $\sigma_{t}^{k}\left(-/-,\;-/+,\;+/-\;\text{or}\;+/-\right)$ through time from the trajectory data $X_{t}^{k}$ (sister *k* = 1, 2). The MCMC algorithm is described in section 1 of S1 Text. The model has an *a posteriori* identifiability problem, i.e., a single trajectory does not have sufficient information to determine all of the parameters; specifically only two of *v*_+_,*v*_−_ and *L* can be inferred from a trajectory because of an (approximate) symmetry in the model (see section 1.3 of S1 Text and S1C Fig). However, this is easily resolved through determination of the spring natural length by treating cells with nocodazole which depolymerises all the spindle MTs, thus allowing the chromatin linkage between sisters to relax (S1A Fig; see also section 3.1 in S1 Text). In absence of external forces (K-fibre forces) the inter-kinetochore distance can be modelled as moving in a harmonic well; thus we were able to infer the rest length of the linkage *L* = 775 ± 110 nm (± population s.d.). This resolved the identifiability problem and enabled the estimation of all parameters in Eq (8) for each paired sister trajectory. Unfortunately we cannot infer the forces directly; only relative forces can be inferred up to the drag coefficient, giving an effective velocity for each force component thereby allowing comparison between these components. This also means that the spring constant *κ* and PEF constant *α* are reported in s^−1^. If the kinetochore/chromatid drag coefficient *γ* is independently determined we can use our method to infer the magnitude of the component forces, nevertheless great insight can be obtained by a comparative analysis alone.

### Reverse engineering individual trajectories

We applied our reverse engineering algorithm for the coherence-incoherence model of kinetochore sister dynamics (Eq 5) to paired sister trajectories derived from 3D live cell imaging of HeLa-K eGFP-CENP-A eGFP-Centrin1 cells. Previous analysis of kinetochore dynamics in human cells using a frame rate of 7.5 s demonstrated a range of stochasticity in individual trajectories with irregular saw-tooth oscillations of approximate period 75 s (10 frames) [8, 17] but the temporal resolution was not sufficient to pinpoint directional switching events. In 2D imaging at a higher frame rate of 2 s kinetochore switches are clearly evident [19] but the window averaging algorithm used to assign P/AP direction in this study was not able to locate switching events. Furthermore, tracking kinetochores in 2D produces short trajectories since kinetochores can move out of the focal plane. To produce suitable data for inferring switching points we used a 3D spinning-disk confocal imaging system capable of a 2 s frame rate with 25 Z-planes (voxel size 138 × 138 × 500 nm^3^) over 5 min and tracked the sub-pixel position of sister kinetochores (Fig 1E; see Burroughs et al. [40]). We observed pseudo-periodic oscillations as previously reported (Fig 1C), with a half-period of 26 s, as determined by autocorrelation analysis. The oscillation is approximately saw-tooth (constant velocity), with clearly defined switching events.

**Fig 2:**
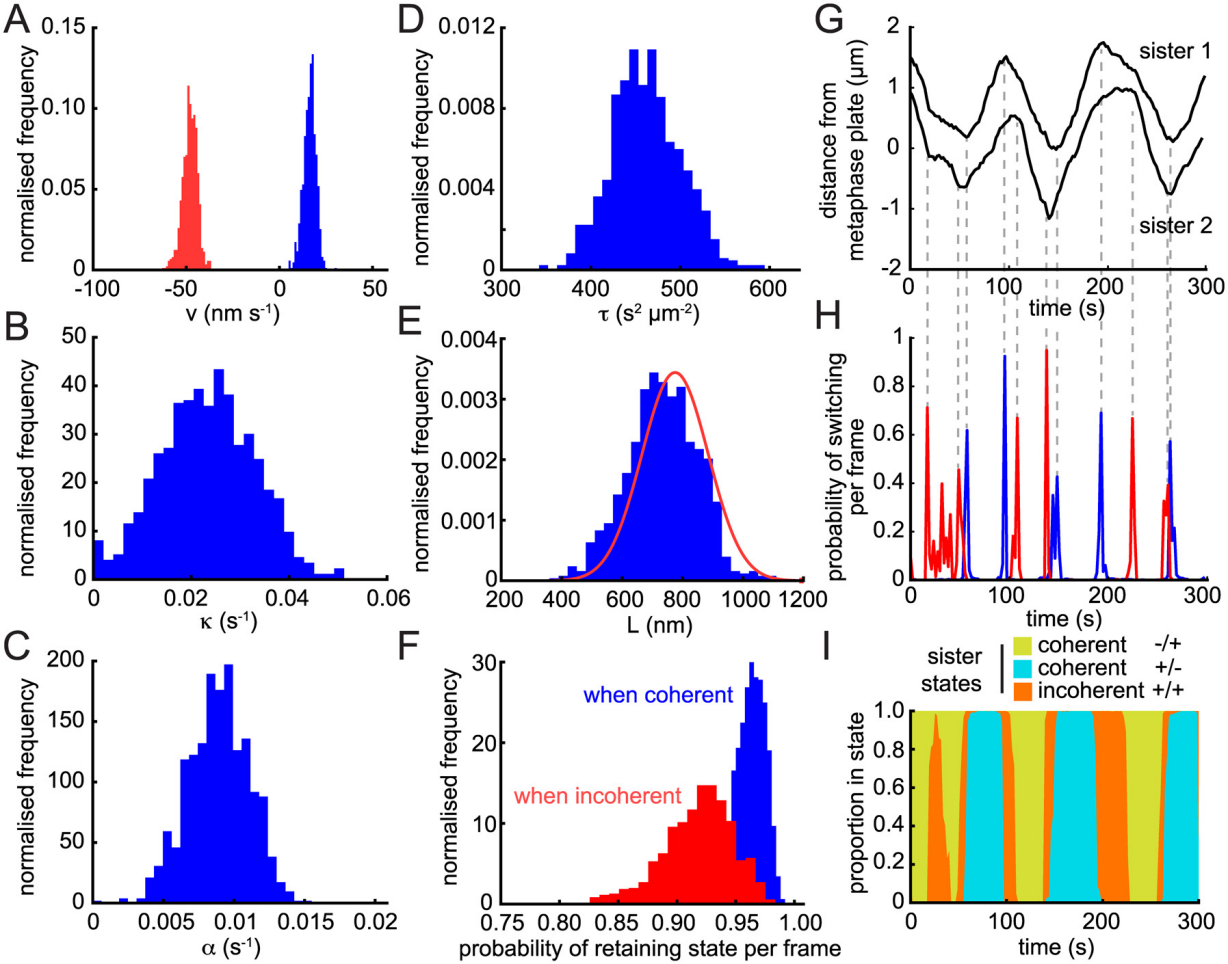
Posterior distribution from a trajectory with strong oscillations. Inferred posterior distributions and model parameters for the single trajectory shown in Fig 1C for (A) the K-fibre (de)polymerisation velocities *v*_+_ (blue) and *v*_−_ (red); (B) spring constant *κ*; (C) anti-poleward force gradient *α*; (D) noise parameter *τ* = *s* ^−2^; (E) natural length *L*; (F) probabilities of not switching state per time-point when sisters are coherent (blue) and incoherent (red). The informed Gaussian prior for *L* determined through nocodazole treatment (see S1 Fig) is shown in red in (E). Posterior distributions consist of over 5,000 samples (see section 1.2 of S1 Text for convergence protocols). (G) Trajectories for sisters of trajectory in Fig 1C with switching points marked by dashed lines. (H) Probability of switching per frame shown for each sister. (I) Posterior probabilities of sister state over the course of the trajectory. Incoherent state –/– does not occur during this particular trajectory.

Parameter estimation and hidden state determination for the trajectory in Fig 1C is illustrated in Fig 2, a trajectory with good oscillatory behaviour. Parameter posteriors appear Gaussian and all show low variance, i.e., low parameter uncertainty (Fig 2A–2F). The natural length *L* posterior is close to the prior; the shift is probably due to the approximate nature of the symmetry, i.e., the twist carries some information. Switching events were confidently identified for both sisters (Fig 2G and 2H). The inferred hidden state shows strong regions of coherence (sisters moving in the same direction) interspersed with short periods of incoherence (sisters moving in opposing directions) that correspond to contraction (+/+) in this trajectory (Fig 2I). There is high confidence in assignment of the sister polymerisation state (Fig 2I) indicative of a highly deterministic behaviour (strong clear oscillations). This particular trajectory shows the previously reported [7] switching choreography wherein the lead sister switches first at every directional switching event. This is also evident directly from the trajectory time-series Fig 1C. This lead sister driven dynamics is responsible for the contracting incoherent state observed between coherent runs and gives the ‘standard’ choreography with the inter-sister distance relaxing at a switching event and increasing over the following half-period as the lead sister moves away [12].

### Identifying oscillatory trajectories using model comparison statistics

It is clear from examining examples of trajectories (S2 Fig) that there is a high degree of variability between kinetochore trajectories. Thus, analysing a few examples is insufficient to assess kinetochore dynamics or the efficacy of a model in capturing those dynamics. We therefore applied our reverse engineering strategy to a large database of trajectories, specifically 1169 sister pairs across 81 cells; on an additional 84 trajectories the MCMC algorithm failed to converge suggesting that the signal-to-noise ratio was low. Visual observation of these trajectories confirmed they were very stochastic; convergence failure was thus a direct consequence of a lack of oscillations. Since our model is aimed at explaining oscillations we could safely ignore non-converging trajectories.

To quantify levels of oscillatory behaviour we used a combination of statistics (see Materials and Methods and section 2 of S1 Text): (i) the explained variance (EV) of the model, which describes how much of the trajectory variance can be explained by the model, (ii) Bayes factors derived from a Bayesian model selection analysis between the coherence-incoherence model and a BM model, and (iii) a directional correlation statistic based on the expected number of directional switches in a BM.

**Fig 3:**
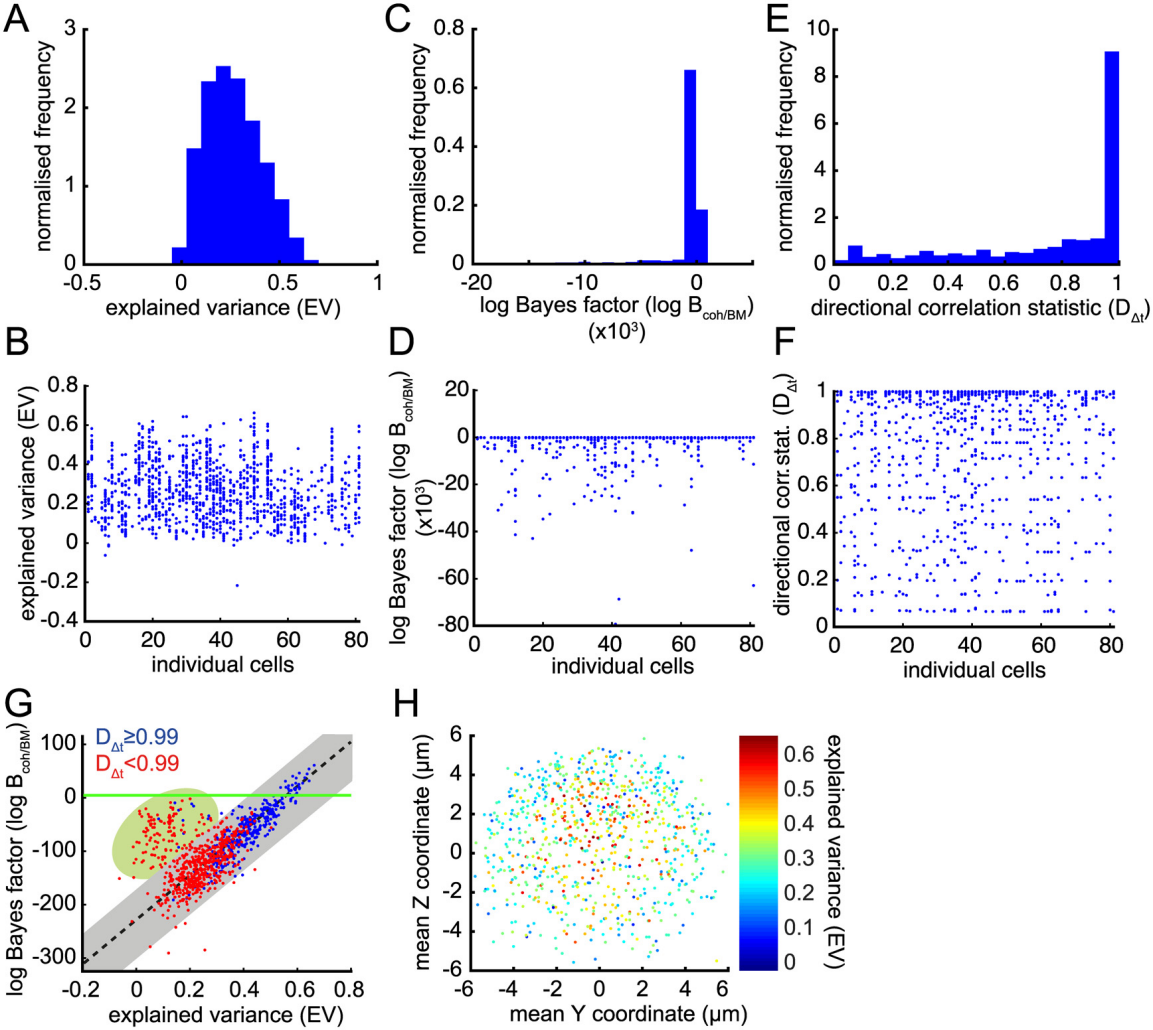
Determination of oscillatory trajectories using three quality statistics. (A) Histogram of explained variance (EV) of each trajectory. (B) Explained variance of each trajectory arranged by cell. (C) Histogram of log Bayes factor of the kinetochore model *M*_coh_ against Brownian motion *M*_BM_ (log *B*[*M*_coh_/*M*_BM_]) of each trajectory. (D) log *B*[*M*_coh_/*M*_BM_] of each trajectory arranged by cell. (E) Histogram of directional correlation statistic *D*_Δ*t*_ of each trajectory. (F) *D*_Δ*t*_ of each trajectory arranged by cell. (G) log *B*[*M*_coh_/*M*_BM_] plotted against EV for each trajectory. Trajectories are coloured according to *D*_Δ*t*_; blue have significant directional correlations. (H) Mean trajectory positions within the metaphase plate viewed along the spindle axis (Y, Z) coloured by EV. All converged trajectories are shown in panels (A-G) (*n* = 1169); only those from grey area in (G) are shown in (H) (*n* = 843).

EV varied from around 0 to 66% (Fig 3A) with mean 26 ± 14% (± distribution s.d.). The variability in trajectories is apparent in the range of EV shown on a per cell basis (Fig 3B), indicating that all cells have a similar profile of near deterministic and highly stochastic trajectories. EV allowed us to rank trajectories by how well the model fitted—the trajectory shown in Fig 1C and reverse engineered in Fig 2 had the highest EV —but does not provide support for the model compared to any other since it is an intrinsic measure of fit. We therefore complemented the EV statistic with a comparative test using the Bayes factor *B*[*M*_coh_/*M*_BM_] between the coherence-incoherence model *M*_coh_ and a Brownian motion (BM) model *M*_BM_ (we also compared against BM models with an inter-sister spring and drift with almost identical results; see section 2.2 of S1 Text and S3A and S3B Fig). Surprisingly, *B*[*M*_coh_/*M*_BM_] showed significant preference for *M*_BM_; we found log *B* [*M*_coh_/*M*_BM_] < 0 for over 95% of the trajectories (Fig 3C and 3D; a log *B*[*M*_coh_/*M*_BM_] < 0 indicates preference for *M*_BM_). This is due to the lack of temporal structure in the *M*_BM_ —displacements are treated as independent, identically Gaussian distributed so *B*[*M*_coh_/*M*_BM_] is predominantly a measure of whether the kinetochore displacements are Gaussian or not. Trajectories where *M*_coh_ is preferred have very regular saw-tooth oscillations and thus an over-representation of large displacements; *B*[*M*_coh_/*M*_BM_] is thus a good discriminator of strong oscillating trajectories. Comparing EV with *B*[*M*_coh_/*M*_BM_] demonstrated a correlation as expected (overall *ρ* = 0.14, *p* = 0.0008; Fig 3G), but also revealed a group of outlier trajectories (green shading in figure) that had higher than average *B*[*M*_coh_/*M*_BM_] but low EV. These trajectories tended to have a few excessively large displacements indicative of tracking errors. We thus filtered these from the analysis by restricting to trajectories approximating the linear relationship between *B*[*M*_coh_/*M*_BM_] and EV (the selection region shown as a grey bar in Fig 3G; *ρ* = 0.90 for grey region) comprising 843 out of 1169 converged trajectories, 72%.

The consistency between EV and *B*[*M*_coh_/*M*_BM_] as measures of oscillatory quality is reassuring; however as discussed above, although able to rank trajectories neither gives a classification into oscillatory and non-oscillatory trajectories, only classifying into Gaussian and non-Gaussian displacements. We therefore used a direction correlation statistic *D*_*n*Δ*t*_ (see section 2.5 of S1 Text) to assess whether higher EV and *B*[*M*_coh_/*M*_BM_] was indicative of correlated displacements as would be expected for an oscillating trajectory which makes repeated sustained runs (Fig 3E and 3F). We found a strong correlation between *D*_1Δ*t*_ and both *B*[*M*_coh_/*M*_BM_] and EV (*r* = 0.49 and 0.57, respectively; S3C and S3D Fig). With subsampling this was drastically reduced, most likely because subsampling reduces noise and thus noisy trajectories, that are penalised under *B*[*M*_coh_/*M*_BM_] or EV (in absence of subsampling), score higher under *D*_*n*Δ*t*_ for *n* > 1. We show trajectories scoring *D*_1Δ*t*_ ≥ 0.99 in blue in Fig 3G (i.e., failing correlation test at 1% significance); the majority of these are in the top-right quadrant indicating that trajectories towards the top-right of the selection region are indeed more oscillatory. All these measures show that all cells have varying levels of stochasticity amongst their trajectories (Fig 3B, 3D and 3F).

The above measures together provide strong evidence that we are able to isolate the oscillatory trajectories (blue in Fig 3G) and these conform to the coherence-incoherence model dynamics, i.e., this model is able to describe the dynamics well. In the remaining sections we limit analysis to those trajectories passing the filtering criterion described above (indicated by grey region in Fig 3G), incorporating both oscillatory and highly stochastic trajectories as determined by the correlation statistic *D*_Δ*t*_.

### Trajectory heterogeneity across the metaphase plate

**Fig 4:**
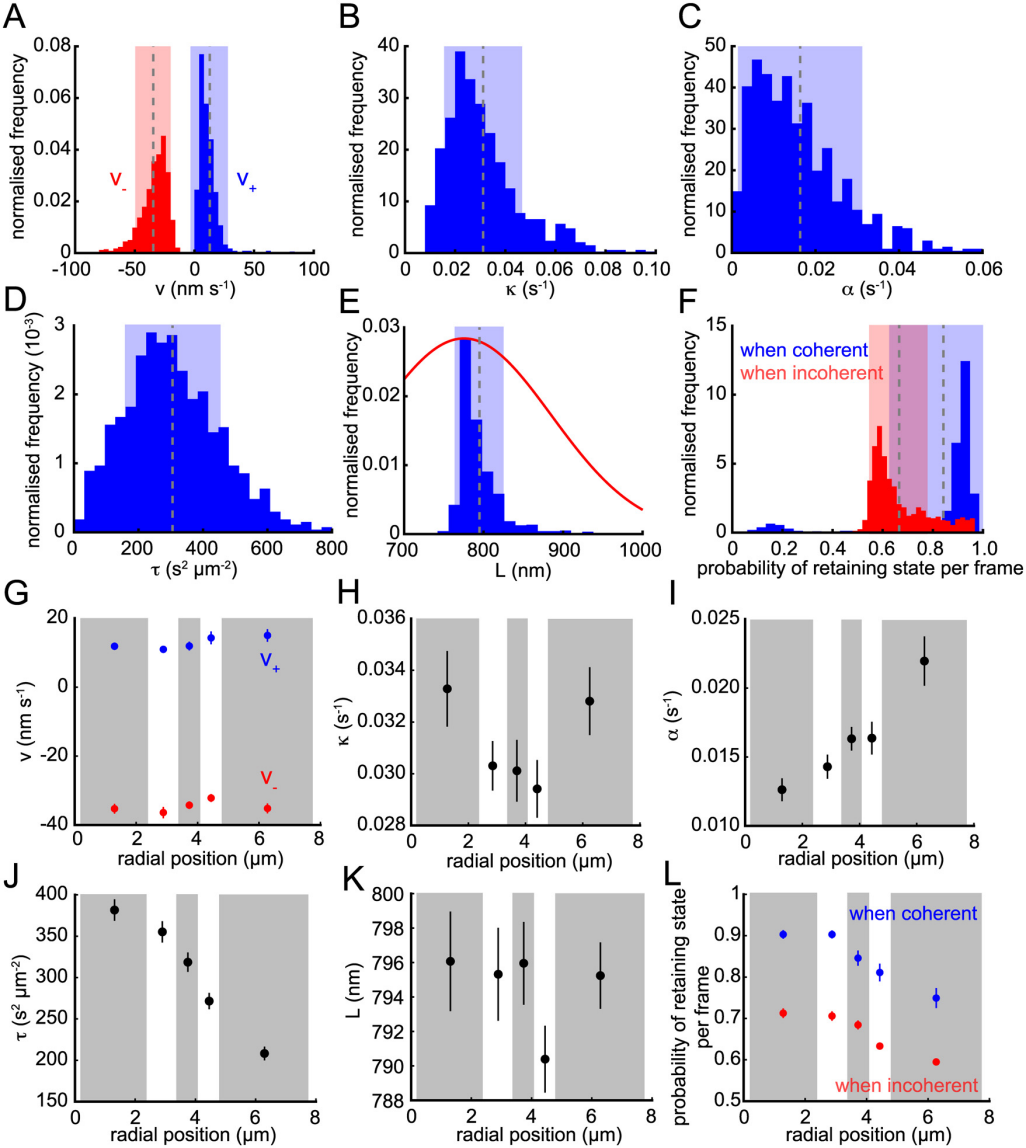
Population parameter analysis. Posterior means for (A) K-fibre (de)polymerisation velocities *v*_+_ (blue) and *v*_−_ (red); (B) spring constant *κ*; (C) anti-poleward force gradient *α*; (D) noise parameter *τ* = *s* ^−2^; (E) natural length *L* (blue); (F) the probabilities of not switching state per time-point when sisters are coherent (blue) and incoherent (red). The informed Gaussian prior on *L* is shown in red in (E). Dashed line and shaded areas in (A-F) indicate mean and ±s.d., respectively. (G-L) Trajectories binned by radial distance from the centre of metaphase plate (with approximately equal numbers per bin; alternating grey and white boxes) for parameters as in (A-F). Circles indicate mean of bin and lines show ±s.d.. All panels include all converged and filtered trajectories (*n* = 843).

As shown in Fig 2, the posterior distributions for the model parameters can be computed for single trajectories, typically giving high confidence parameter estimates. To summarise across the dataset, we used the posterior means of each trajectory (Fig 4); these show that there is significant heterogeneity between trajectories in their parameter values, with ranges over an order of magnitude in many cases. The natural length is the exception because it is constrained by the informed nocodazole prior and thus the means are tightly clustered (mean 795 ± 31 nm; Fig 4E) about the prior mean (775 ± 110 nm). These parameter estimates can be used to unravel the forces driving kinetochore movements as follows.

It is widely believed that the depolymerising K-fibre attached to the leading sister provides the dominant driving force in kinetochore motion [33, 43]. Our data supports this view with the estimate for *v*_−_ being significantly larger in magnitude than *v*_+_ (*v*_−_ = −35 ± 15 vs. *v*_+_ = 13 ± 16 nm s^−1^, ± s.d., with ∣*v*_−_∣ > *v*_+_ in 97% of trajectories, *p* < 10^−202;^ binomial test); recall speed is commensurate with force in our model and *v*_±_ are the inferred speed components. This indicates that depolymerising forces are significantly larger than polymerising (Fig 4A). Typical kinetochore speeds are around 23 ± 6 nm s^−1^, similar to the average of *v*_+_ and ∣*v*_−_∣ [8], whilst ∣*v*_−_∣ > *v*_+_ implies that the sisters will typically separate over the course of a coherent run (standard choreography), increasing the inter-sister distance. This is consistent with the measured average inter-sister distance of around 1 µm, which means that the inter-kinetochore spring is typically under tension with an average extension of 205 ± 31 nm.

For the spring and PEF, we found that *κ* ≈ 2*α* (Fig 4B and 4C) implying that the PEF is equal in magnitude to the inter-kinetochore spring force when kinetochores are twice as far from the metaphase plate as the spring is extended; thus the PEF typically dominates the spring force at displacements away from the metaphase plate of over 0.5 μm. This also indicates that the PEF is effective at maintaining a thin metaphase plate since for displacements of a couple of µm the PEF is comparable to the force from the K-fibre, although the linear approximation for the PEF may lose validity at displacements over this distance [37].

Fig 4F shows the estimated probabilities of staying coherent, *p*_*c*_ (blue), and staying incoherent, *p*_*ic*_ (red), between consecutive frames, with mean frame lengths of 6.3 ± 8.7 and 3.0 ± 1.0 for coherent and incoherent periods. The strong skew towards 1 of *p*_*c*_ demonstrates that coherent runs are stable; it is relatively unlikely to switch states between consecutive frames giving rises to extended runs. Naturally, this effect is less pronounced for noisier trajectories since they would be expected to make fewer coherent runs or switch more often (S3E and S3F Fig).

The distribution of the noise precision *τ* (= *s* ^−2^) showed a range from close to zero to 800 s^2^ μm^−2^ (where larger numbers indicate less noise; Fig 4D). Our analysis of trajectory stochasticity had already shown that individual cells harbour inter-trajectory heterogeneity (Fig 3B, 3D and 3F). Kinetochore pairs located nearer the centre of the cell had more oscillatory trajectories as judged by EV (Fig 3H), consistent with previous observations that oscillatory trajectories tend to be more centrally located within the metaphase plate [44]. To ascertain if this heterogeneity is also reflected in the dynamic parameters we explored parameter trends with respect to distance from the centre of the metaphase plate *r* (Fig 4G–4L). The most significant trends were with regard to the trajectory noise (*ρ* = −0.43, *p* < 10^−37^; Fig 4J) and PEF strength coefficient (*ρ* = 0.20, *p* < 10^−8^; Fig 4I) which almost doubles between the centre and periphery. The former correlates with stochasticity as measured by EV (Fig 3H), *τ* and EV being highly correlated (*ρ* = 0.57, *p* < 10^−73^). This increase in stochasticity with *r* also explains the increase in the probability of switching direction per frame with *r* (*ρ* = −0.27, *p* < 10^−14^ when coherent, *ρ* = −0.37, *p* < 10^−28^ when incoherent; Fig 4L), kinetochore movements becoming more random and liable to switch direction per frame. There was no significant dependence on metaphase plate position for the other parameters: *v*_±_, *L* and *κ*.

### Relative force components during oscillations

**Fig 5:**
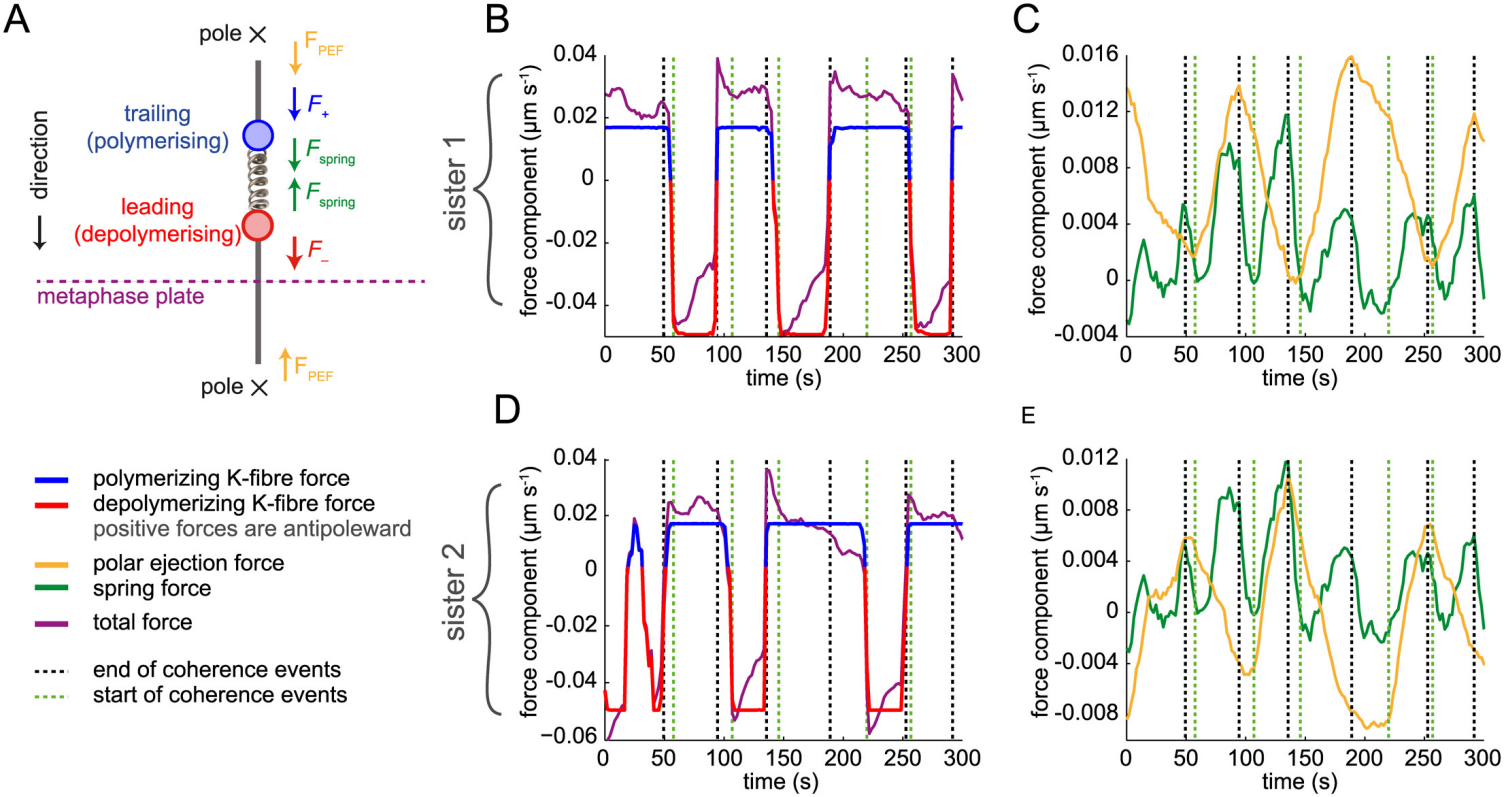
Force decomposition profile. (A) Schematic of the forces acting on kinetochore sisters. (B-E) Estimated decomposition of kinetochore velocity into its force components over time for the trajectory in Fig 1C; (B, D) sister 1 and (C, E) sister 2. Polymerisation/depolymerisation force on kinetochores attributable to the K-fibre (blue/red, respectively); spring force (green); polar ejection forces (orange) and the total force (purple). Time-series overlaid with inferred end (black dashed) and start (green dashed) times of coherence periods (runs). Between end and start times K-fibres are incoherent (in this case +/+) Note that forces are positive in the anti-poleward direction.

From the parameter estimates and inferred K-fibre state determined for each trajectory, we can use Eq (3) to calculate effective contributions to the total force on each kinetochore (recall that in our model the parameterisation of forces and velocities are commensurate; we quote forces in μm s^−1^). Specifically we can estimate the contributions of the 3 component forces: the PEF, the K-fibre forces and the spring force (Fig 5A) throughout the trajectory. In Fig 5B and 5C we show the forces acting on one sister (sister 1) of the sister pair of Fig 1C; Fig 5D and 5E show the same for the second sister (sister 2). The K-fibre force on sister 1 was by far the dominant force on the kinetochores and because it corresponds to the K-fibre state, it changes sign at directional switches (Fig 5B). The PEF and spring force were a minor contribution, and were in phase every period, the spring force having double the period of the oscillation as previously observed [8, 12]. A comparison between the sisters (Fig 5B and 5D) clearly demonstrates the approximate anti-phase of the K-fibre state, although this is not exact and extended periods of incoherence did occur; in this example, both sisters have polymerising K-fibres during incoherence. This is reflected in the spring force profile (Fig 5C and 5E) since +/+ incoherence causes relaxation of the spring to near zero spring tension and apparent compression (*F*_spring_ < 0) during the extended period of incoherence. The PEF force, being positional and linearised in our model, corresponds to the kinetochore position thereby reflecting the off-plate position of the sister pair (Fig 1C).

#### Directional switching analysis

**Fig 6:**
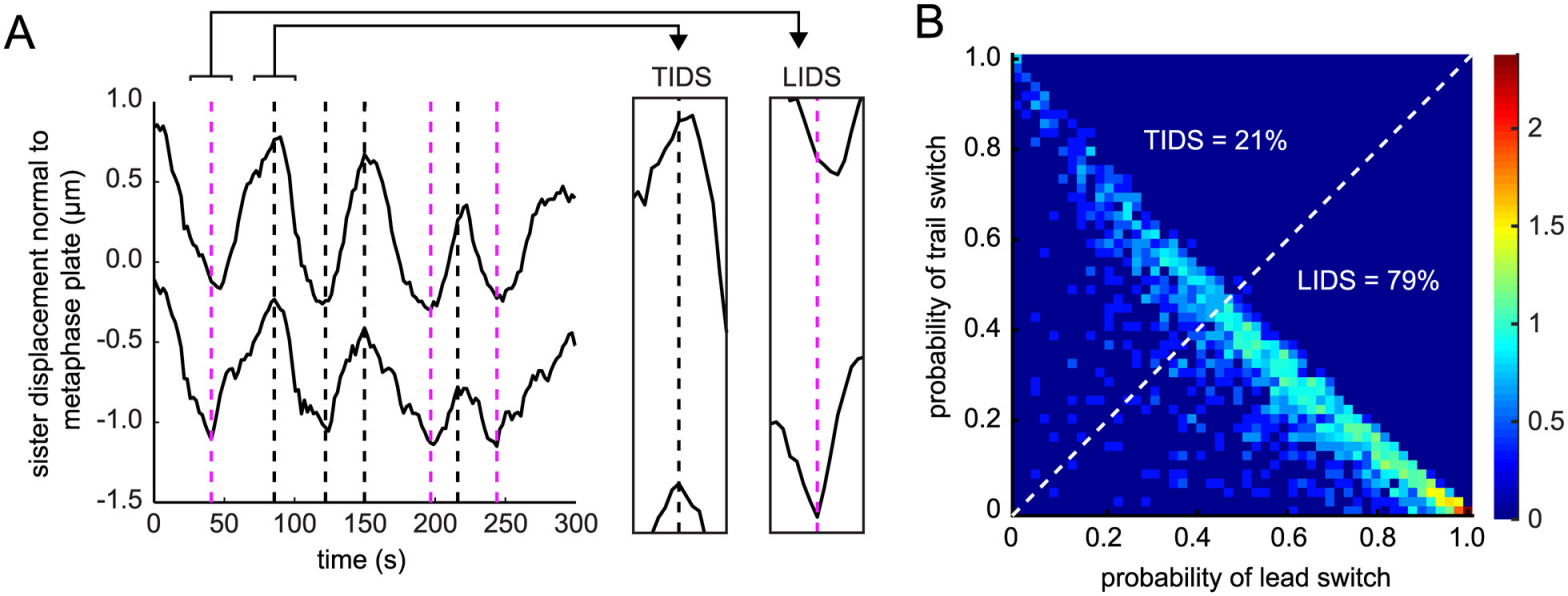
Lead or the trailing sister can initiate directional switching. (A) Example trajectory exhibiting lead initiated directional switching (LIDS; magenta dashed lines) and trail initiated directional switching (TIDS; black dashed lines). (B) Probability of the trailing vs. lead sister switching first (n = 2063 switch events). Dotted white line indicates the boundary where *p*_LIDS_ = *p*_TIDS_.

In addition to inferring the force parameters, our method also estimates the probability of each K-fibre being in a + or − state at each time-point ($\sigma_{i}^{k}$). From these state probabilities we estimated the switching times for going into and out of coherent runs (the pair of states +/– or –/+; see example of Fig 6A)—these correspond to switches in direction. We used coherent runs to define switching since the oscillations are comprised of periods of coherence separated by a directional switching event. We defined coherent runs in $\sigma_{i}^{k}$ as a consecutive sequence of at least 5 time points in the same direction, although we allow for transient changes of direction because of noise (see Materials and Methods). The end of these coherent runs then defines the initiation of a directional switch. As trajectory stochasticity increases, coherent runs become less frequent; as expected we saw an increase in the number of detected switches as EV increases from 025% (S3E Fig). However, paradoxically the number of switches fell again for trajectories with EV > 25%.

This can be explained by considering two oscillating trajectories, one of which contains more directional reversals, i.e., when a sister changes direction, but then changes back to the original direction. The trajectory with more reversals has more switch events and, moreover, is more Brownian since the correlation between successive displacements is reduced. This is reflected in the negative correlation between the number of switches and *B*[*M*_coh_/*M*_BM_] (S3F Fig).

For each coherent run we can compute the probability of the lead sister or the trailing sister ending the run. More often than not the leading sister switched before the trailing sister (Fig 6B), as expected from previous reports in Ptk1 cells [12]. However, the priority of the lead sister was not exclusive, and we also observed the reverse choreography—the trailing sister switching first (Fig 6A), previously observed in Ptk1 cells [9] suggesting that this is a common phenomena of metaphase oscillations. We call these events a Leading Sister Initiated Directional Switch (LIDS) and a Trailing Sister Initiated Directional Switch (TIDS) respectively, and we classify events by the largest probability of the respective sister switching, *p*_LIDS_ > *p*_TIDS_ for the leading sister, *p*_LIDS_ < *p*_TIDS_ for the trailing sister switching first. Over the population of trajectories we found a strong bias for LIDS events, 79% (1614) of the total switches, with only 21% (449) being TIDS events (Fig 6B).

#### Kinetochore switching is not a result of a tug-of-war between opposing forces

**Fig 7:**
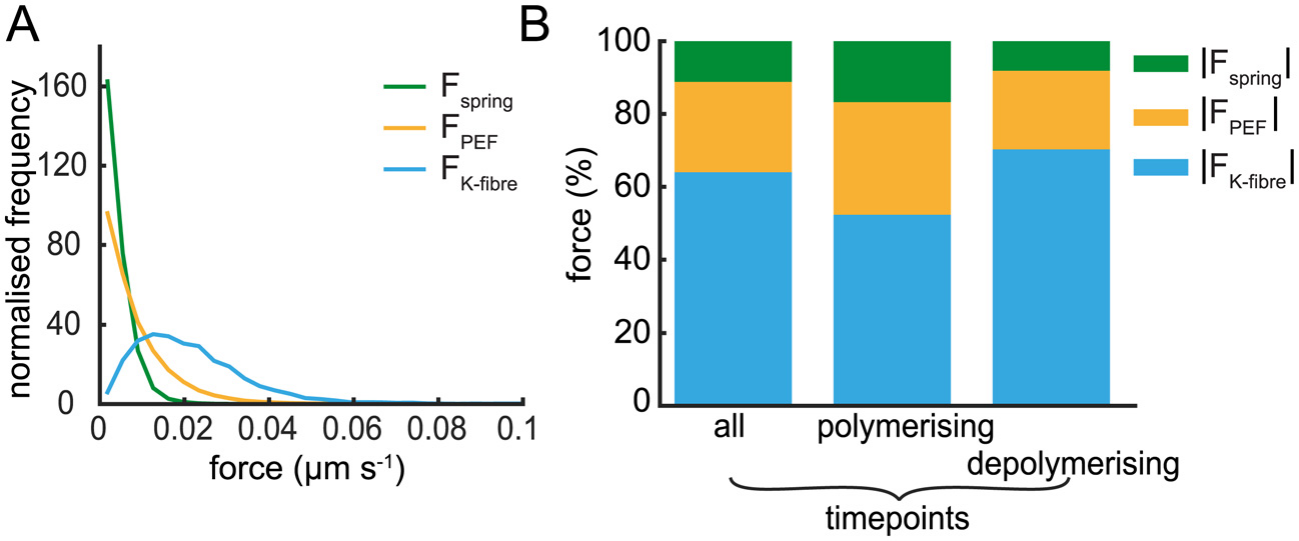
Average force decomposition across the population of trajectories. (A) Distributions of absolute forces (spring, PEF and K-fibre) averaged over trajectories (and sisters). (B) Mean force partition averaged over all time-points, time-points where the kinetochore is attached to a polymerising or depolymerising K-fibre. Absolute force values are averaged over respective sister and time-points across all trajectories (*n* = 843).

**Fig 8:**
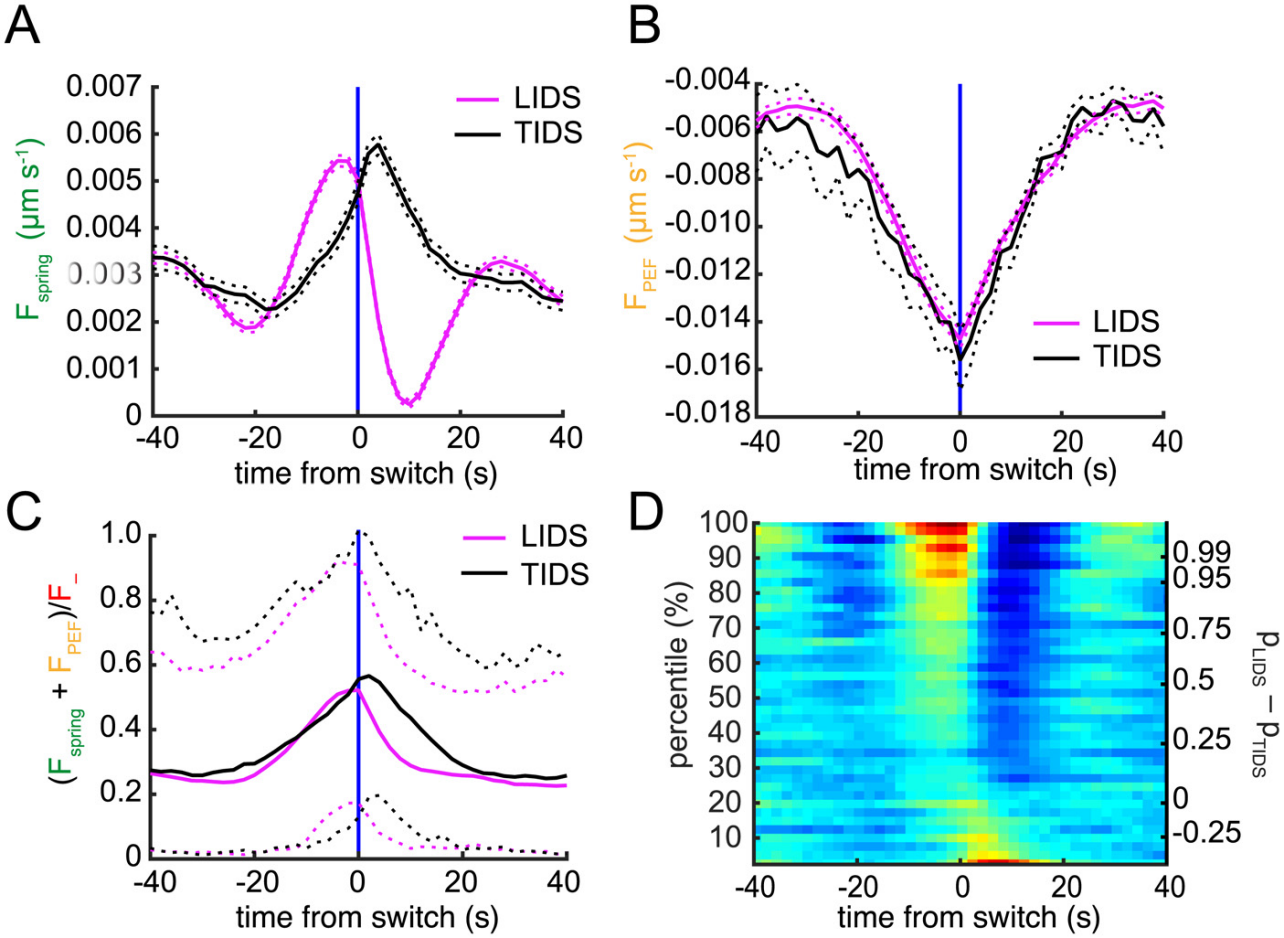
Force profiles during directional switches. Time-dependent estimation of (A) spring force and (B) PEF. Switch events from trajectories where the lead (LIDS, *n* = 1614; magenta) or trail (TIDS, *n* = 449; black) kinetochore switched first were aligned at their median switching time (the time origin is marked by vertical blue line). Solid lines indicate mean, dashed lines ±s.e.m.. Forces are given as velocities by rescaling by the viscosity coefficient. (C) Opposing force (spring + PEF) on lead sister during a LIDS (magenta) or TIDS (black) is plotted relative to the depolymerisation force *F*_−_. Solid lines indicate mean, dashed lines 5% and 95% percentiles of the population. (D) Spring force heat map across switching events partitioned by difference between probability of LIDS (*p*_LIDS_) or TIDS (*p*_TIDS_). Events above zero on right *y*-axis are classified as LIDS; those below as TIDS.

To obtain insight into the forces governing LIDS and TIDS events we computed the average contribution of each force component throughout the trajectory. The distributions of these forces clearly show that the spring force is the lowest contributor, followed by the PEF, while the force from the K-fibre is the largest contribution (Fig 7A). The K-fibre forces comprise 64% of the combined (absolute) forces on average (Fig 7B), with the spring force being only 11% on average (17%, 8% during poleward, antipoleward movements, respectively). However, these forces vary dynamically over the trajectory (see Fig 5). We therefore used the detected switching time as a temporal fiducial marker to align force profiles from multiple switch events. We performed this procedure after separating events into LIDS and TIDS thereby obtaining an averaged profile for each event type. Although the spring force was the least in magnitude during the run (Fig 7A), it had the strongest pre-switch signature discriminating between LIDS and TIDS (Fig 8A). The PEF profile was identical regardless of which sister switched first (Fig 8B), although there was a tendency for TIDS to occur at a slightly increased distance from the metaphase plate. For LIDS, the spring force increased significantly in the 20 s leading up to the switch, peaking at around -4 s with an increase of over 150% relative to that at -20 s (Fig 8A). This high spring tension (deduced in prior studies from changes in the inter-sister distance) has been suggested as the trigger for lead sister switching [7, 12, 45, 46]. During a LIDS both K-fibres are in a polymerising state, since the previously depolymerising lead sister switches first, which causes a reduction in spring force immediately post-switch (Fig 8A). This loss of tension is thought to be the trigger for the switch of the second sister [7, 12, 45, 46]. Conversely, for a TIDS an increase of the spring force occurs post-switch as the two sisters are attached to depolymerising K-fibres, stretching the centromeric spring (Fig 8B); this stretch relaxing over 20 s post-switch. This high tension 2 s post-switch could trigger the initially leading sister to switch thereby completing the directional switch of the two sisters. However, TIDS events cannot be explained by the tension arguments above; the spring tension (and inter-sister stretch) reach their maximum after the trailing sister has switched so a tension trigger on the trailing sister cannot be the cause of a TIDS event. Another mechanism must be in place to cause the trailing sister to switch first. Examining the force contributions across events indicates that the spring force, despite its high level of change across the switching event, remains a minor component rising to at most 20% during a LIDS. In fact, examination of the opposing forces (sum of PEF and spring force) to the depolymerising K-fibre attached to the leading sister indicate that they are rarely sufficient to stall the leading sister, only resisting on average 50% of the pulling force at the switch (Fig 8C).

The heatmap in Fig 8D shows the switch force profiles binned into percentiles according to *p*_LIDS_−*p*_TIDS_, that is, the difference in probability of a switch being LIDS or TIDS. The top row averages events with very strong evidence in favour of a LIDS classification; the bottom similarly for TIDS. This method of visualisation reveals a continuum of switching profiles from a strong LIDS signature with high spring force building up prior to the switch, the weakening of this profile through to the threshold *p*_LIDS_ = *p*_TIDS_ and the emergence of the TIDS profile with increased spring force post-switch.

#### PEF heterogeneity organises the metaphase plate

**Fig 9:**
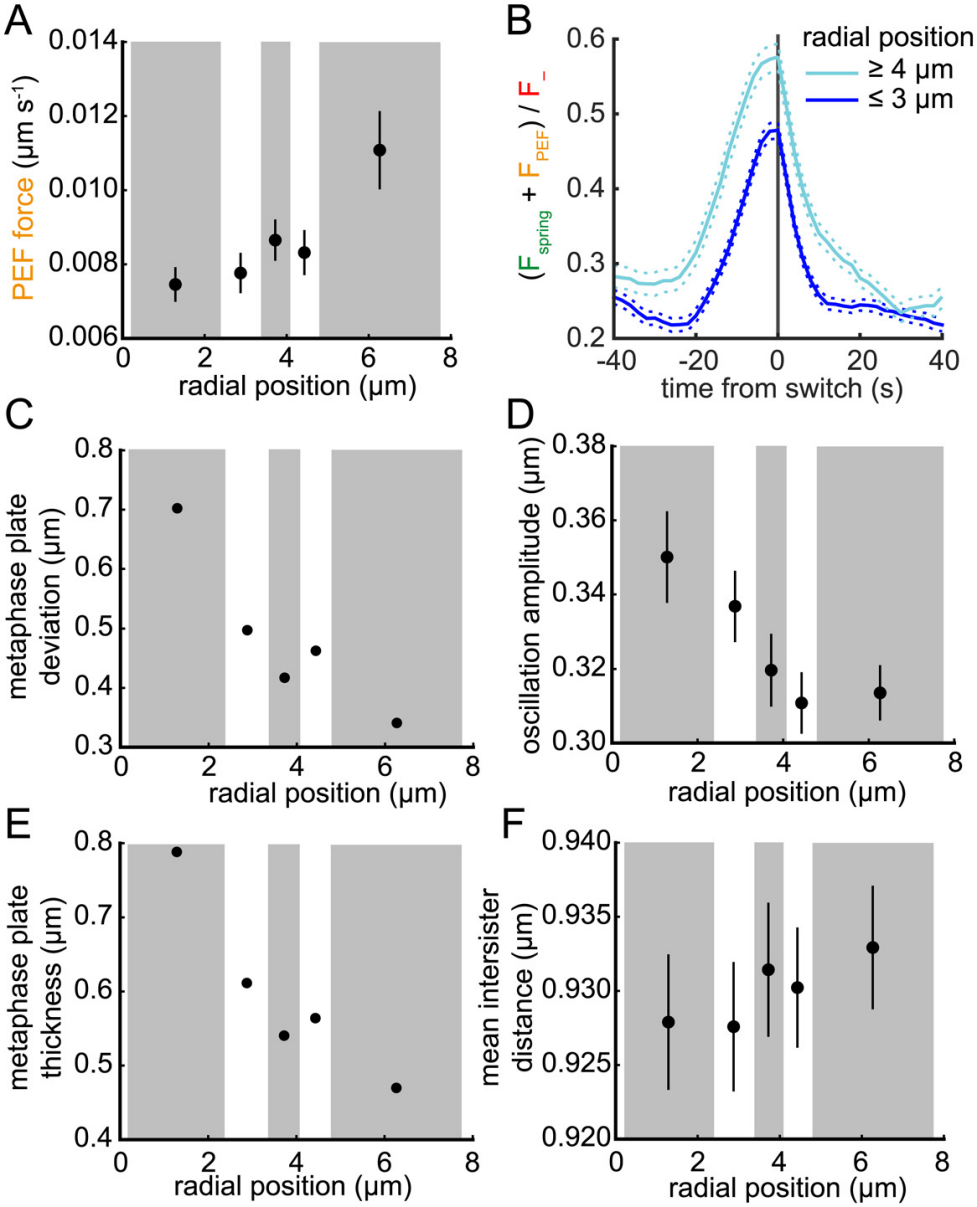
PEF variation over the metaphase plate impacts oscillation amplitude and deviation from alignment. (A) Average absolute PEF (on sister 1) binned by distance *r* from the centre of the metaphase plate (bins of approximately equal number; alternating grey and white boxes). (B) Average profile of the proportion of the opposing force (spring + PEF) to *F*_−_ on lead sister during a LIDS for trajectories with *r* ≥ 4 μm (cyan) and *r* ≤ 3 μm (blue). Switch events aligned as described under Fig 8. Solid and dashed lines indicate mean and ±s.e.m., respectively. (C-F) Trajectories binned by distance *r* as in (A) with bin mean of (C) alignment deviation; (D) oscillation amplitude; (E) metaphase plate thickness; (F) inter-sister distance shown. Lines in (D,F) indicate s.d., *n* = 843, see text for definitions.

Our analysis revealed that the PEF strength coefficient *α* increased with distance *r* from the centre of the metaphase plate (Fig 4I). We expect this to have an impact on the oscillatory dynamics, i.e., the average PEF force should be higher at the spindle periphery although it also depends on the degree to which the oscillation amplitude decreases in response to a more constraining PEF potential. In fact, a higher PEF is expected to both decrease the oscillation amplitude and improve the alignment of the sisters at the metaphase plate. We measure the latter with the alignment deviation (defined as the standard deviation of the distribution of time averaged sister mid-points 〈(*X*_1_ + *X*_2_)/2〉) and quantify the oscillation amplitude with the standard deviation of the sister mid-point trajectory; in essence this is a between and within variance analysis of the sister mid-point (ANOVA). We show in Fig 9A that the average PEF does indeed increase with *r* (*ρ* = 0.13, *p* < 10^−4^), although less than the PEF coefficient (Fig 4I) with only a 50% increase towards the periphery. The dependence on *r* was also evident in profiles of the opposing force (PEF + spring force) to the leading sister; however, even at the periphery of the plate stalling is atypical (Fig 9B). Excursions away from the plate decreased under the higher PEF potential at increased *r* with both a decrease in the alignment deviation and oscillation standard deviation (Fig 9C and 9D). The finite length of the trajectories inflates both these measures because of incomplete periods within each trajectory. We also confirmed that the higher PEF at the periphery caused the metaphase plate to thin using the plate thickness statistic of Jaqaman et al. [8] (the standard deviation of the pooled sister mid-point positions over time and trajectories; Fig 9E). In part the reduced oscillation amplitude contributes to the increased trajectory stochasticity since the oscillatory signal relative to noise is reduced, potentially explaining the reduction in *τ* towards the periphery (inversely proportional to noise; Fig 4J).

## Discussion

In this paper we have presented a framework for the (Bayesian) reverse engineering of individual paired kinetochore trajectories utilising a mathematical model of kinetochore oscillations. This tool enables a whole swathe of new analyses to be performed across a range of levels, specifically: (i) The processing of kinetochore tracks, locating switch points and partitioning trajectories into coherent and incoherent phases, thereby facilitating interpretation of this complex, stochastic dynamical system. (ii) A high-throughput semi-automated analysis at the level of populations of kinetochore trajectories, generating a range of population level statistics. There is no typical trajectory since there is both a high variability in trajectory stochasticity (here and [8]) and heterogeneity within cells, evidenced by significant parameter trends across the metaphase plate (Fig 4) that are only evident upon analysis of 1000s of trajectories. (iii) Force inference and identification of mechanistic signatures, thereby revealing previously inaccessible mechanistic information. Our methodology complements forward simulation analyses (reviewed in [33, 34, 47]) where models of kinetochore dynamics are developed from knowledge and assumptions pertaining to the biological processes and are demonstrated to qualitatively or semi-quantitatively reproduce observed behaviour under suitable parameter values. However, our analysis of 2 second resolution 3D live-cell tracking data has revealed new depths of complexity in kinetochore dynamics and choreography, substantially adding to the qualitative and quantitative constraints that these models must satisfy, including trail sister initiated switching, a bias towards lead sister initiated switching, and spatial parameter trends. Reproducing these probabilistic behaviours is likely to require the incorporation of new mechanistic or regulatory processes. Moreover, we have demonstrated that data-driven reverse engineering methods are able to fit biologically meaningful mechanistic models to real trajectory data. Developing more realistic mechanistic models within this framework is the next challenge; the model selection methods we have implemented here will be invaluable in determining whether mechanistic hypotheses are supported by data.

The kinetochore dynamics model we have proposed here is parsimonious, only incorporating the three essential forces believed to affect kinetochore positioning, whilst simplifying the form of these forces. Firstly, we have treated each K-fibre coarsely as producing a single unified force. K-fibres are bundles of microtubules and therefore both the number of microtubules attached to the kinetochore varies [48] and the balance between the number of polymerising and depolymerising microtubules will change as they individually undergo catastrophe and rescue events [19]. How kinetochores control the dynamic instability of the attached microtubules, thereby giving rise to K-fibres that are either polymerising or depolymerising remains a mystery. Incorporating this level of complexity is not possible within the reverse engineering framework since individual kinetochore trajectory dynamics do not have enough information to determine the K-fibre composition. However, since there is typically a majority of either polymerising or depolymerising microtubules within each K-fibre [19, 42], the coarse-grained model is a reasonable approximation. The composition fluctuations, that are likely to produce deviations from this constant force approximation will inflate the noise in the fits of our model to data. In the future, it may be possible to combine quantification of fluorescent tubulin (number of microtubules) and fluorescent EB proteins (fraction of polymerising microtubules [19]) proximal to the kinetochore with trajectory data to incorporate these effects into the reverse engineering. Secondly, we have assumed that the PEF is linear around the metaphase plate. This is well-supported experimentally for the size of displacements typical of metaphase kinetochores [37]. Incorporating a nonlinear form for the PEF (stronger at the poles) is feasible, although it is likely that kinetochore trajectories that explore locations closer to the spindle poles will be needed to obtain reliable estimates of that nonlinearity. This would be true in prometaphase, where kinetochores congress to the plate from all over the mitotic spindle, and may be a crucial change necessary for the model to account for this phase. Thirdly, we have assumed that the centromeric spring is a linear (Hookean) spring. Recently, it has been suggested that this spring is nonlinear [49] and by incorporating this into the reverse engineering model it may be possible to determine the nature of the nonlinearity and any asymmetry in the spring. More complex models, such as visco-elastic models are likely beyond the scope of what is identifiable with trajectory data alone.

As it stands the model is simple enough to allow parametrisation from individual trajectories subject to additional data on the natural length of the inter-sister spring being supplied. Our reverse engineering approach using this model allows behavioural and mechanistic (force) variability amongst trajectories to be quantified at a previously inaccessible level, in particular spatial trends could be detected in the force components across the metaphase plate (Fig 4G–4L, Fig 9). Our analysis of the quality of the fit indicates that kinetochore oscillations are well-explained by this simple force balance model, with explained variance (EV) reaching as high as 66%, similar to levels achieved on simulated data using estimated parameter values (not shown). A minority of trajectories show very strong preference for this model over a simple Brownian motion model (Fig 3C, 3D and 3G). This indicates that displacements at the 2 second level retain a strong signature of the underlying dynamics, although as trajectory stochasticity increases the displacements lose that signature and conform to a Gaussian distribution. Correlations between consecutive displacements are however retained for the majority of trajectories, even for highly stochastic trajectories (Fig 3G), suggesting that directional switching of K-fibres drives kinetochore motion even when highly stochastic.

Reverse engineering is able to provide crucial biological insight into complex, heavily regulated systems such as kinetochore dynamics. Firstly, directional switching has two choreographies, lead initiated directional switching (LIDS) and trail initiated directional switching (TIDS), with the latter inhibited in the eGFP-CENP-A/eGFP-Centrin1 cell line examined here (bias 4:1, Fig 6B). Secondly, the spring force is weak, constituting typically less than 15% of the total force acting on a kinetochore and on average rising only to 20% at switching. Even combined with the PEF, which both oppose the pulling force on the lead kinetochore by its K-fibre, a stall in the lead kinetochore under force equilibration is extremely rare (Fig 8C). This means that kinetochore switching is not a result of a tug-of-war between opposing forces (as various models predict [35, 50], and as occurs in *Drosophila* [51]). In effect, both kinetochores move at 13 to 35 nm s^−1^ with directional switching events essentially preventing the inter-sister distance increasing too high, thereby keeping the spring tension low. This suggests that tension is utilised as a means to regulate switching, as suggested in [7, 12, 45, 46], to both prevent occurrence of a high spring tension—which could potentially cause separation of the sister chromatids—and to localise the kinetochore at the plate. Thirdly, by aligning many hundreds of switching events, we revealed a distinct switching signature which provides experimental validation of the lead-sister induced switch model [7, 12, 45, 46]. Using the inferred model parameters, we estimate the relative contributions of each force component prior to and across the switching events. This clearly revealed the spring force as the minor force component throughout the profile (Figs 7 and 8); kinetochore dynamics are dominated by K-fibre forces. The increase in spring force leading up the directional switch most likely triggers the switch [7, 12, 45, 46]. However, a substantial revision of these ideas is required to unify this LIDS mechanism with the alternative TIDS choreography (see [40]). Fourthly, as previously reported for Ptk1 cells [44], the most strongly oscillating trajectories are located in the centre of the metaphase plate (Fig 3H). We show that there are concomitant changes in the switching rates with distance from the centre (Fig 4L), and an increase in the PEF (Figs 4I, 9A and 9B) correlates with increased plate thinning with distance from the centre of the metaphase plate and damping of oscillations (Fig 9C–9E). This is in agreement with a laser microsurgery study which demonstrated that reducing the PEF increases the amplitude of oscillations [37] and concurs with the proposal that the PEF is the cause for the loss of oscillations towards the plate periphery in [44].

Here we quantified the inferred forces in terms of speed (μm s^−1^). This is because we have no reliable estimate of the effective drag coefficient *γ* of the kinetochore/chromatid system; non-thermal contributions to noise from mechanical fluctuations imply estimation from *τ* would result in significant underestimation. If it were possible to robustly determine *γ* experimentally, we would be able to separate the current *τ* into intrinsic active and external molecular stochasticity, and directly determine the forces. To obtain estimates of absolute forces we can appeal to a simple approximation using Stokes’ law [3], assuming a chromosome radius of 0.5 μm (estimated from volume measurements [52]) and a spindle viscosity of 190 Pa s[53]. This yields mean absolute forces for the three mechanical components of *F*_−_ = 62 pN, *F*_+_ = 23 pN, *F*_spring_ = 6.9 pN, and *F*_PEF_ = 15 pN. The calculated force from a P-moving kinetochore (*F*_−_; depolymerising) is compatible with the 50 pN stall force measured by Nicklas in pre-anaphase cells [2]. This indicates that our reverse engineering is extracting physiologically meaningful forces.

Our reverse engineering approach raises significant questions. Firstly, how is kinetochore directional switching regulated to generate the observed pseudo-periodic oscillations incorporating both LIDS and TIDS choreographies, with TIDS being inhibited relative to LIDS. Secondly, how is this switching accomplished under low spring tension, that rarely, if ever, achieves a kinetochore stall. Thirdly, what changes in the spindle explain the increase in trajectory stochasticity and the PEF with distance from the centre of the metaphase plate. Future work is needed to tie down the biochemical and mechanical processes that underpin these behaviours. We expect reverse engineering will be invaluable in this endeavour to both quantify how the behaviour changes with genetic perturbations and also for fitting increasingly complex models of the kinetochore-microtubule interface.

## Supporting Information

S1 Text Markov chain Monte Carlo algorithm, model selection and explained variance statistic. Extended methods for live-cell imaging and kinetochore tracking.
(PDF)

S1 Fig Analysis of identifiability of *L*. (A) Distribution of posterior means of the natural length L from MCMC sampling using a 1D harmonic well model for cells treated with nocodazole. Trajectories filtered to those with EV > 0.25. (B) Posterior (solid) distribution of *L* for 4 different priors (dashed), for a typical trajectory. Each prior is a truncated Gaussian distribution (reported means and standard deviations refer to Gaussian distributions before truncation). (C) Fractional difference in parameter posterior mean for trajectories with EV > 0.25, with prior mean of *L* altered by -20%, -10%, 10% and 20%. * and ** represent significant difference in Mann-Whitney test at 5% and 1% levels, respectively.
(EPS)

S2 Fig Exemplar trajectories with different noise characteristics. A random selection of trajectories from three regions of (A) Bayes factor *B*[*M*_coh_/*M*_BM_] and explained variance EV plot (reproduced from Fig 3G) are shown in (B). The top row have high EV and high *B*[*M*_coh_/*M*_BM_]; the middle have low EV and low *B*[*M*_coh_/*M*_BM_]; the bottom have high *B*[*M*_coh_/*M*_BM_] but low EV.
(EPS)

S3 Fig Analysis of trajectory quality. Log marginal likelihood of (A) *M*_spring-BM_ against *M*_BM_ and (B) *M*_spring-drift-BM_ against *M*_BM_. Correlation (Pearson’s) between directional correlation statistic *D*_*n*Δ*t*_ and (C) *B*[*M*_coh_/*M*_BM_]; or (D) EV, for different subsample rates *n*Δ*t*. Number of switch events detected in each trajectory plotted by (E) EV; (F) *B*[*M*_coh_/*M*_BM_]. Red solid line indicates mean number of events after binning into 10 bins; dashed lines indicate ±s.d.. All converged trajectories are used in panels (A-D) (*n* = 1169); only those from grey area in 3G are used (*n* = 843).
(EPS)
