# Supplementary material for: Inferring the Forces Controlling Metaphase Kinetochore Oscillations by Reverse Engineering System Dynamics
Source: PLoS Comput Biol. 2015 Nov 30;11(11):e1004607. doi: 10.1371/journal.pcbi.1004607 (PMC4664287; doi:10.1371/journal.pcbi.1004607)
Supplement: S1 Text — Extended methods for live-cell imaging and kinetochore tracking. (PDF) [file pcbi.1004607.s001.pdf]

Supplementary Text for Inferring the Forces Controlling Metaphase Kinetochore Oscillations by Reverse Engineering System Dynamics

Jonathan W. Armond, Edward F. Harry, Andrew D. McAinsh, Nigel J. Burroughs

Contents

1 Markov chain Monte Carlo algorithm **2**
1.1 Parameter updates . . . 2
1.1.1 Precision τ . . . 2
1.1.2 Velocities $v_{\pm}$ . . . 2
1.1.3 Spring constant κ . . . 3
1.1.4 Spring natural length L . . . 3
1.1.5 Anti-poleward force parameter α . . . 3
1.1.6 State transition probabilities p_c, p_{ic} . . . 4
1.1.7 Hidden states σ_t^k . . . 4
1.2 Initial conditions, mixing and convergence . . . 4
1.3 Identifiability . . . 5

2 Model selection details **5**
2.1 Brownian motion model . . . 5
2.2 Brownian motion models with spring and drift . . . 5
2.3 Marginal likelihoods . . . 5
2.4 Explained variance . . . 6
2.5 Directional correlation statistic . . . 6

3 Live cell imaging and kinetochore tracking **7**
3.1 Nocodazole treatment for spring rest length determination . . . 7

1 Markov chain Monte Carlo algorithm

Table 1. Prior parameters used for Markov chain Monte Carlo inference.

Parameter	Prior parameters	
p_c	α_{c}=2.5	β_{c}=1
p_{ic}	α_{ic}=2	β_{ic}=1
τ	c=0.5	d=0.001
L	μ_L=0.775	s_L^2=0.0121
κ	μ_κ=0.05	s_κ^2=10000
$v_\pm$	$\mu_{v_\pm}$=±0.03	$s_{v_\pm}^2$=10
α	μ_α=0.01	s_α^2=10000

1.1 Parameter updates

We update each parameter separately by drawing samples from the full conditional probability distributions as follows:

1.1.1 Precision τ

The conditional distribution $\pi(\tau|\cdot)$ is Gamma distributed, where $\pi(\cdot)$ indicates a probability function and the conditioning is on all parameters and data except τ, giving the update

$$\tau|\cdot \sim \Gamma(c + n - 1, d + \frac{1}{2}\sum_{k=1}^{2}\sum_{i=1}^{n-1}(x_{i+1}^k - x_i^k - f_i^k)^2) \tag{1}$$

where $f_i^k(\sigma_i^k) = (-1)^k v_{\sigma_i^k} + (-1)^k \kappa(d_i - L\cos(\theta_i)) - \alpha x_i^k$, and parameters are defined as in main text.

1.1.2 Velocities $v_\pm$

The conditional distributions on $v_\pm$ are Gaussian distributed. For v_+ we have conditional

$$\begin{aligned}\pi(v_+|\cdot) \propto \exp\Bigg[-\frac{1}{2}\Bigg(&\frac{1}{s_v^2}(v_+ - \mu_v)^2 + \\ &\tau\sum_{k=1}^{2}\sum_{\substack{1\le i<n,\\ \sigma_i^k=+}}\left[x_{i+1}^k - x_i^k - (-1)^k v_+ - (-1)^k\kappa(x_i^1 - x_i^2 - L\cos(\theta_i)) + \alpha x_i^k\right]^2\Bigg)\Bigg]\end{aligned} \tag{2}$$

and similarly for v_-. Completing the square, we have for $v_\pm$

$$v_\pm|\cdot \sim \mathcal{N}\left(\frac{1}{\tau_v^\pm}\left[\pm\mu_v s_v^{-2} + \tau\sum_{k=1}^{2}\sum_{\substack{1\le i<n,\\ \sigma_i^k=\pm}}\left[(-1)^k(x_{i+1}^k - x_i^k + \alpha x_i^k) - \kappa(x_i^1 - x_i^2 - L\cos(\theta_i))\right]\right], \frac{1}{\tau_v^\pm}\right) \tag{3}$$

with $\tau_v^\pm = \tau n_\pm + s_v^{-2}$. Here $n_\pm$ are the number of time points in state $\pm$ respectively combined over both sister trajectories and we have $n_+ + n_- = 2(n-1)$. We use a Metropolis-Hasting rejection step to impose the constraints $v_+ > 0, v_- < 0$, using the above as a proposal.

1.1.3 Spring constant κ

The conditional distribution on κ is Gaussian. It is given by

$$\pi(\kappa|\cdot) \propto \exp\left[-\frac{1}{2}\left(\frac{1}{s_\kappa^2}(\kappa-\mu_\kappa)^2 + \tau\sum_{k=1}^{2}\sum_{i=1}^{n-1}\left[x_{i+1}^k - x_i^k - (-1)^k v_{\sigma_i^k} - (-1)^k\kappa(x_i^1 - x_i^2 - L\cos\theta_i) + \alpha x_i^k\right]^2\right)\right] \tag{4}$$

Completing the square gives the update

$$\kappa|\cdot \sim \mathcal{N}\left(\left[\mu_\kappa s_\kappa^{-2} + \tau\sum_{k=1}^{2}\sum_{i=1}^{n-1}\left((-1)^k(x_{i+1}^k - x_i^k + \alpha x_i^k) - v_{\sigma_i^k}\right)(x_i^1 - x_i^2 - L\cos\theta_i)\right]/\tau_\kappa,\ \tau_\kappa^{-1}\right) \tag{5}$$

with $\tau_\kappa = s_\kappa^{-2} + 2\tau\sum_{i=1}^{n-1}(x_i^1 - x_i^2 - L\cos\theta_i)^2$. We impose the constraint $\kappa \geq 0$ by using the above as a proposal with a Metropolis-Hasting rejection step.

1.1.4 Spring natural length L

The natural spring length L has conditional

$$\begin{aligned}\pi(L|\cdot) \propto \exp\Bigg[-\frac{1}{2}\Bigg(&\frac{1}{s_L^2}(L-\mu_L)^2 + \\ &\tau\sum_{k=1}^{2}\sum_{i=1}^{n-1}\left(x_{i+1}^k - x_i^k - (-1)^k v_{\sigma_i^k} - (-1)^k\kappa(x_i^1 - x_i^2 - L\cos(\theta_i)) + \alpha x_i^k\right)^2\Bigg)\Bigg]\end{aligned} \tag{6}$$

Completing the square gives the update

$$L|\cdot \sim \mathcal{N}\left(\left[\mu_L s_L^{-2} + \tau\kappa\sum_{k=1}^{2}\sum_{i=1}^{n-1}\cos(\theta_i)\left((-1)^{k+1}(x_{i+1}^k - x_i^k + \alpha x_i^k) + v_{\sigma_i^k} + \kappa(x_i^1 - x_i^2)\right)\right]/\tau_L, \tau_L^{-1}\right) \tag{7}$$

with $\tau_L = s_L^{-2} + 2\tau\kappa^2\sum_{i=1}^{n-1}\cos^2(\theta_i)$. Again, we impose positivity with a rejection step.

1.1.5 Anti-poleward force parameter α

The conditional $\pi(\alpha|\cdot)$ is a Gaussian,

$$\begin{aligned}\pi(\alpha|\cdot) \propto \exp\Bigg[-\frac{1}{2}\Bigg(&\frac{1}{s_\alpha^2}(\alpha-\mu_\alpha)^2 + \\ &\tau\sum_{k=1}^{2}\sum_{i=1}^{n-1}\left(x_{i+1}^k - x_i^k + (-1)^{k+1} v_{\sigma_i^k} + (-1)^{k+1}\kappa(x_i^1 - x_i^2 - L\cos\theta_i) + \alpha x_i^k\right)^2\Bigg)\Bigg]\end{aligned} \tag{8}$$

with $\alpha > 0$. This gives a conditional posterior,

$$\alpha|\cdot \sim N\left((\mu_\alpha s_\alpha^{-2} - \tau\sum_{k,i} x_i^k\left(x_{i+1}^k - x_i^k + (-)^{k+1}v_{\sigma_i^k} + (-)^{k+1}\kappa(x_i^1 - x_i^2 - L\cos\theta_i)\right))/\tau_\alpha, \tau_\alpha^{-1}\right) \tag{9}$$

with $\tau_\alpha = s_\alpha^{-2} + \tau\sum_{i=1}^{n-1}\sum_{k=1}^{2}(x_i^k)^2$ and truncated to $\alpha > 0$ by rejection step.

1.1.6 State transition probabilities p_c, p_{ic}

The conditionals on p_c, p_{ic} are Beta distributed as follows

$$p_c \Big| \cdot \sim Beta(a_c + N_c - K_c, b_c + K_c) \tag{10}$$

where N_c, K_c are the number of coherence time points and the number of switches to incoherence respectively for the (current) sister hidden states σ_t^k. Similarly,

$$p_{ic} \Big| \cdot \sim Beta(a_{ic} + N_{ic} - K_{ic}, b_{ic} + K_{ic}) \tag{11}$$

1.1.7 Hidden states σ_t^k

The hidden MC can be updated by a Gibbs move locally at each time point [1]. Define a_{rs} as the transition matrix between the states $(+,-)$, with p_c, p_{ic} the probabilities of no change, a depending on the current state. The conditional for state σ_i^k, sister k is then given by

$$\pi(\sigma_i^k = r|\cdot) \propto a_{\sigma_{i-1}^k r}(\sigma_{i-1}^{\hat{k}})\phi(\Delta X_i^k|\sigma_i^k = r, \cdot)a_{r\sigma_{i+1}^k}(\sigma_i^{\hat{k}})$$

where ϕ is the Gaussian pdf of ΔX_i^k depending on σ_i^k, and we indicate the dependence of a on the other sister $\hat{k}$ (determining whether the sisters are coherent/incoherent). The first and last time points are slightly different, as there is no preceding/following state. This conditional is easily normalised for a Gibbs update, running consecutively over sisters and through time.

However, this algorithm has poor mixing; on the Gelman-Rubin convergence statistic [2] the noise τ is the slowest to converge because of poor convergence of the hidden MC. Performance is improved by using two moves. Firstly, doing a joint update of both sisters at each time point, i.e. we use the above conditional (product over sisters) to compute the probabilities of states $(+,+),(+,-),(-,+),(-,-)$ at each time point and use a Gibbs update. Secondly, we use a block move of length w for each sister separately. This is computationally time consuming taking about 4 times longer than the joint sister update. We found a 20% mix of the block move, w randomly selected over 4-10 gave as good convergence as a full block move.

The block move is constructed as follows. Define row vectors u_0, u_1 associated with current states 0,1 at position i. Then iteratively move to the next time point reconstructing

$$u_r = [u_0 a_{0r}\phi(\Delta X_i^k|\sigma_i^k = 0, \cdot)] \oplus [u_1 a_{1r}\phi(\Delta X_i^k|\sigma_i^k = 1, \cdot)]$$

for sister k, thereby doubling the length of u_r. This works through the window. Initialise the vectors by $u_r = a_{sr}$ if $\sigma_{i_1}^k = s$, and then u_r is the unnormalised probability vector of all 2^w states if $\sigma_{i_{w+1}}^k = r$. The state in the window is then updated using a Gibbs move. Windows from the start of the time series have a prior on the initial position as above. Computational costs to update over the full time series are similar for $w = 2...11$, thereafter rising.

1.2 Initial conditions, mixing and convergence

We ran 5 chains for each sister pair from over-dispersed initial conditions and used the Gelman-Rubin statistic [2] to test for convergence. Chains were initially run up to 250,000 samples and sub-sampled by a factor of 20. If they failed to converge (convergence diagnostic > 1.2 on any parameter), run length was doubled (subsampling frequency also doubled), repeated up to a maximum of 3 times to meet the convergence criterion. Any trajectory with a convergence statistic still above 1.2 was considered unconverged and discarded from further analysis. The hidden state was initialised from a simulation of the MC given the initial parameters p_c, p_{ic}. We used the last 5000 samples per chain for final analysis, discarding burn-in.

1.3 Identifiability

The model in (5) has an identifiability problem for constant twist θ_t, i.e., data in a single trajectory is insufficient to determine all the parameters. This derives from the symmetry $L \to L + l/\cos\theta$, $v_\pm \to v_\pm + \kappa l$ for arbitrary l; thus only the two combinations $v_+ - v_-$, $v_+ - \kappa L \cos\theta$ (and recombinations of these) are independent of the symmetry; thus the model is *a priori* unidentifiable. The sign constraints $v_+ > 0$ and $v_- < 0$ limit this identifiability symmetry to $-v_+/\kappa < l < -v_-/\kappa$ in addition to $L + l/\cos\theta > 0$, where L and $v_\pm$ are the unknown true values. This symmetry is lost if θ_t varies over the trajectory; however the low variation of θ_t leaves an *a posteriori* identifiability problem where the posterior distribution for L shifts towards higher values as the prior variance is increased (Fig. S1B). To infer the model we therefore require an informed prior on L; using a prior for L determined from nocadozole treated cells (Fig. S1A) we found all other parameters were estimatable from the data. We tested the sensitivity of estimated parameters to changes in the L prior mean (Fig. S1C) and found that apart from $v_\pm$ (which was expected due to the nature of symmetry) almost all other parameters were robust, κ being the next most sensitive showing significant change only when the L prior mean was decreased by 20%. This confirmed that any error in determining the natural length (because of other effects of nocadozole treatment, such as compaction of the chromosomes) did not significantly affect our conclusions as regards $v_\pm$, and did not affect conclusions pertaining to the other parameters at all.

2 Model selection details

2.1 Brownian motion model

Brownian motion in an over-damped environment (with negligible inertia) can be modelled by the following stochastic differential equation

$$dX_t = \sqrt{2D}\, dW_t \tag{12}$$

where D is the diffusion coefficient. Integrating over a small time interval Δt gives the approximate displacement, to order $\mathcal{O}(\Delta t)$

$$X_{t+\Delta t} = X_t + \eta_t \Delta t \tag{13}$$

where $\eta_t \sim \mathcal{N}(0, s^2)$ and $s^2 = 2D/\Delta t$. Hence, frame-to-frame displacements are approximately

$$\frac{X_{t+\Delta t} - X_t}{\Delta t} = \frac{\Delta X}{\Delta t} = \eta_t \sim \mathcal{N}(0, s^2) \tag{14}$$

For two sisters (X^1,X^2), the likelihood of a set of displacements $\{\Delta x_i^k\}$ is

$$\pi(\{\Delta x_i^k\}|\tau) = \frac{\tau^{n-1}}{(2\pi)^{n-1}} \prod_k \exp\left(-\frac{\tau}{2}\sum_{i=1}^{n-1}(\Delta x_i^k)^2\right) \tag{15}$$

where $\tau = s^{-2}$ is called the precision.

2.2 Brownian motion models with spring and drift

We also computed the Bayes factor of M_{coh} against two other Brownian models: one with sister pairs connected by a Hookean spring but otherwise each undergoing a 1D BM, $M_{\text{spring-BM}}$, and further extension of $M_{\text{spring-BM}}$ to allow for a constant drift acting on both sisters, $M_{\text{spring-drift-BM}}$. The Bayes factors for these models largely agreed with that for M_{BM} (Fig. S3A,B) so we did not compare these models further.

2.3 Marginal likelihoods

To compare the support for different models we computed marginal likelihoods to estimate Bayes factors, that is, the posterior odds ratios conditioned on two models. We employed algorithms due to Chib [3] and Chen [4] to take advantage of previously generated MCMC samples. Chib's method also requires generating

further chains with blocks of the parameters fixed. We sequentially fixed each parameter in each subsequent chain, setting the fixed parameter to its posterior mean estimate. The extra chains incur considerable additional computational cost, therefore after checking that the difference in marginal likelihood between the two methods was negligible over a wide range of trajectories, we favoured Chen's method, which is based on the following identity for K samples from a converged MCMC chain

$$\pi(\mathbf{X}|M) = \log \pi(\mathbf{X}|\mathbf{\Theta}^*) - \log \left[\frac{1}{K} \sum_{k=1}^{K} \frac{g(\mathbf{\Theta}^{(k)}|\mathbf{\Sigma}^{(k)})}{\pi(\mathbf{\Theta}^*)} \frac{\pi(\mathbf{\Theta}^*|\mathbf{\Sigma}^{(k)}, \mathbf{X})}{\pi(\mathbf{\Theta}^{(k)}|\mathbf{\Sigma}^{(k)}, \mathbf{X})} \right] \tag{16}$$

where $\mathbf{X}$ is the displacement data, $\mathbf{\Theta}^*$ is a fixed point for the model parameters (here, the posterior mean estimate), $\mathbf{\Theta}^{(k)}$ and $\mathbf{\Sigma}^{(k)}$ are parameter and hidden state samples from the MCMC run, respectively and $g(\mathbf{\Theta}_k|\mathbf{\Sigma}^{(k)})$ is an arbitrary density. To calculate $\pi(\mathbf{X}|\mathbf{\Theta}^*)$ we integrated out the hidden state $\mathbf{\Sigma}$ by importance sampling weighted by drawing samples from the posterior distribution for $\mathbf{\Sigma}$. The choice of g affects the variance of the estimate. We set g to the product of Γ distributions fitted independently to the posterior samples for parameters $v_\pm$, κ, α, L, and τ, and Beta distributions similarly fitted for p_c, p_{ic}. This choice ensures that the density of g will not deviate too far from the joint posterior density. We tested that the resulting marginal likelihood estimate was independent of this choice by comparing with estimates derived using $g(\mathbf{\Theta}|\mathbf{\Sigma}) = \pi(\mathbf{\Theta})$ and estimating the variance of both methods using overlapping batch statistics [4]. The variance under the fitted g was markedly lower but the mean estimates were consistent across trajectories. Having computed the marginal likelihoods for two models M_1 and M_2, their Bayes factor is $B_{1/2} = \pi(\mathbf{X}|M_1)/\pi(\mathbf{X}|M_2)$ and the data supports M_1 over M_2 when $B_{1/2} > 1$.

2.4 Explained variance

The explained variance (EV) is a measure of how well a model accounts for the variance of a trajectory. Given the model parameters, hidden state and data we can compute the sum of squares

$$V[X] = \sum_{k=1,2} \sum_{i=1}^{n} (x_{i+1}^k - x_i^k - f_i^k(\sigma_i^k))^2 \tag{17}$$

where f_i^k is as in (7). Here V is the difference between the predicted displacements of the model and the observed displacements. By averaging over the posterior samples from the MCMC, we obtain the average explained variance,

$$\text{EV} = 1 - \frac{\mathbf{E}_{posterior}[V]}{\text{Var}_0} \tag{18}$$

where

$$\text{Var}_0 = \sum_{k=1,2} \sum_{i=1}^{n} (x_{i+1}^k - x_i^k)^2 \tag{19}$$

is an estimate of the displacement variance of the two sisters. This is equal to the displacement variance if we subtract the mean displacement but this is close to zero for long time series.

2.5 Directional correlation statistic

In a Brownian motion the probability of a change in direction of motion (i.e., $\text{sign}(\Delta x_i) \neq \text{sign}(\Delta x_{i+1})$) is 0.5 and is independent at each time-step. Hence the number of directional switches K in n time-points is binomially distributed

$$P(K = k) = \binom{n-2}{k} \frac{1}{2^{n-2}}. \tag{20}$$

The number of switches k in a trajectory is computed as the number of times the sign of the displacement of the centre position between the two sisters changes (i.e., $\text{sign}(\Delta(x_i^1 + x_i^2))$). We also examined subsampled displacements over the interval $n\Delta t$. Using this we define a statistic

$$D_{n\Delta t} = 1 - 2\min(P(K \leq k), P(K > k)) \in [0, 1] \tag{21}$$

A trajectory is not likely to be Brownian motion if $D_{n\Delta t} \approx 1$ due to correlations between successive displacements.

3 Live cell imaging and kinetochore tracking

HeLa-Kyoto (K) cells stably expressing eGFP-CENP-A / eGFP-Centrin1 were cultivated and maintained as described in [5]. Cells were seeded in 35 mm glass bottom dishes (MatTek Corporation) and the media changed to Leibovitz L-15 supplemented with 10% foetal calf serum prior to imaging. Cells were imaged using a 100X 1.4 NA oil objective on a confocal spinning-disk microscope (VOX Ultraview; Perkin and Elmer) with a Hamamatsu ORCA-R2 camera, controlled by Volocity 6.0 (Perkin and Elmer). Mitotic cells were first identified using bright-field illumination to minimise phototoxicity. Image stacks (25 Z-sections, 0.5 µm spacing) were collected every 2 seconds for 5 minutes (150 time-points per video). Camera pixels were binned 2×2 giving an effective pixel size of 138 nm in the lateral direction with 16 bits per pixel imaging depth. Exposure conditions were 50 ms per Z-slice using a 488 nm laser set to 15% power. 90% of cells entered anaphase after measurement demonstrating that cells were unharmed by phototoxicity. Image time-series were deconvolved using Huygens 4.1 (SVI) using a point-spread function measured from micro-bead images. Kinetochores trajectories were tracked as previously described [6] except we implemented a 3D Gaussian mixture model algorithm for refining spot positions to sub-pixel accuracy [7], based on an earlier 2D version [8]. MATLAB kinetochore tracking software (KiT) is available from `http://mechanochemistry.org/mcainsh/software.php` and will be described in detail in a forthcoming paper.

3.1 Nocodazole treatment for spring rest length determination

To estimate the spring rest length, we treated eGFP-CENP-A / eGFP-Centrin1 HeLa-K cells with nocodazole. This causes depolymerisation of all microtubules, thus relieving kinetochore pairs of tension. Cells were imaged in prometaphase as described in section 3, except with 31 Z-sections and 0.2 µm spacing. Higher Z resolution was necessary because kinetochores pairs were randomly oriented in 3D. Kinetochore trajectories were tracked as described in section 3.
To accurately determine the spring rest length, we fitted trajectories to a 1D harmonic oscillator model

$$D_{t+\Delta_t} - D_t = -\kappa(D_t - L) + N(0, s^2) \tag{22}$$

where D_t is the distance between sister kinetochores at time t, κ is the spring constant and L is the rest length. s^2 quantifies the noise in the trajectory. We used an analogous MCMC algorithm to section 1, excluding any need to compute hidden states, to infer L from individual trajectories.

References

1. Robert CP, Celeux G, Diebolt J. Bayesian estimation of hidden Markov chains: A stochastic implementation. Stat Probab Lett. 1993;16:77–83.
2. Gelman A, Rubin DB. Inference from Iterative Simulation Using Multiple Sequences. Stat Sci. 1992;7(4):457–511.
3. Chib S. Marginal Likelihood from the Gibbs Output. J Am Stat Assoc. 1995;90(432):1313–1321.
4. Chen MH. Computing marginal likelihoods from a single MCMC output. Stat Neerl. 2005;59(1):16–29.
5. Vladimirou E, Mchedlishvili N, Gasic I, Armond JW, Samora CP, Meraldi P, et al. Nonautonomous movement of chromosomes in mitosis. Dev Cell. 2013;27(1):60–71.
6. Jaqaman K, King EM, Amaro AC, Winter JR, Dorn JF, Elliott HL, et al. Kinetochore alignment within the metaphase plate is regulated by centromere stiffness and microtubule depolymerases. J Cell Biol. 2010;188(5):665–679.

7. Burroughs NJ, Harry EF, McAinsh AD. Super-resolution kinetochore tracking reveals the mechanisms of human sister kinetochore directional switching. Elife. 2015;10.7554/eLife.09500.

8. Thomann D, Rines DR, Sorger PK, Danuser G. Automatic fluorescent tag detection in 3D with super-resolution: Application to the analysis of chromosome movement. J Microsc. 2002;208(1):49–64.
